# Expanded Proteomic Survey of the Human Parasite Leishmania major Focusing on Changes in Null Mutants of the Golgi GDP-Mannose/Fucose/Arabinopyranose Transporter *LPG2* and of the Mitochondrial Fucosyltransferase *FUT1*

**DOI:** 10.1128/spectrum.03052-22

**Published:** 2022-11-17

**Authors:** Gloria Polanco, Nichollas E. Scott, Lon F. Lye, Stephen M. Beverley

**Affiliations:** a Department of Molecular Microbiology, Washington University School of Medicine, St. Louis, Missouri, USA; b Department of Microbiology and Immunology, University of Melbourne at the Peter Doherty Institute for Infection and Immunity, Melbourne, Victoria, Australia; University of Illinois at Urbana Champaign

**Keywords:** N-linked glycans, N-linked glycoconjugates, trypanosomatid protozoan parasite, fucose, glycoproteome, kinetoplast, mitochondria, ultradeep proteomics

## Abstract

The trypanosomatid protozoan parasite *Leishmania* has a significant impact on human health globally. Understanding the pathways associated with virulence within this significant pathogen is critical for identifying novel vaccination and chemotherapy targets. Within this study we leverage an ultradeep proteomic approach to improve our understanding of two virulence-associated genes in *Leishmania*, encoding the Golgi mannose/arabinopyranose/fucose nucleotide-sugar transporter (*LPG2*) and the mitochondrial fucosyltransferase (*FUT1*). Using deep peptide fractionation followed by complementary fragmentation approaches with higher-energy collisional dissociation (HCD) and electron transfer dissociation (ETD) allowed the identification of over 6,500 proteins, nearly doubling the experimentally known Leishmania major proteome. This deep proteomic analysis revealed significant quantitative differences in both Δ*lpg2*^–^ and Δ*fut1*^s^ mutants with *FUT1*-dependent changes linked to marked alterations within mitochondrion-associated proteins, while *LPG2*-dependent changes impacted many pathways, including the secretory pathway. While the FUT1 enzyme has been shown to fucosylate peptides *in vitro*, no evidence for protein fucosylation was identified within our ultradeep analysis, nor did we observe fucosylated glycans within *Leishmania* glycopeptides isolated using hydrophilic interaction liquid chromatography (HILIC) enrichment. This work provides a critical resource for the community on the observable *Leishmania* proteome as well as highlighting phenotypic changes associated with *LPG2* or *FUT1*, ablation of which may guide the development of future therapeutics.

**IMPORTANCE** *Leishmania* is a widespread trypanosomatid protozoan parasite of humans, with ~12 million cases currently, ranging from mild to fatal, and hundreds of millions asymptomatically infected. This work advances knowledge of the experimental proteome by nearly 2-fold, to more than 6,500 proteins and thus provides a great resource to investigators seeking to decode how this parasite is transmitted and causes disease and to identify new targets for therapeutic intervention. The ultradeep proteomics approach identified potential proteins underlying the “persistence-without-pathology” phenotype of mutants with deletion of the Golgi nucleotide transporter LPG2, showing many alterations and several candidates. Studies of a rare mutant with deletion of the mitochondrial fucosyltransferase FUT1 revealed changes underlying its strong mitochondrial dysfunction but did not reveal examples of fucosylation of either peptides or *N*-glycans. This suggests that this vital protein’s elusive target(s) may be more complex than the methods used could detect or that this target may not be a protein but perhaps another glycoconjugate or glycolipid.

## INTRODUCTION

**FIG 1:**
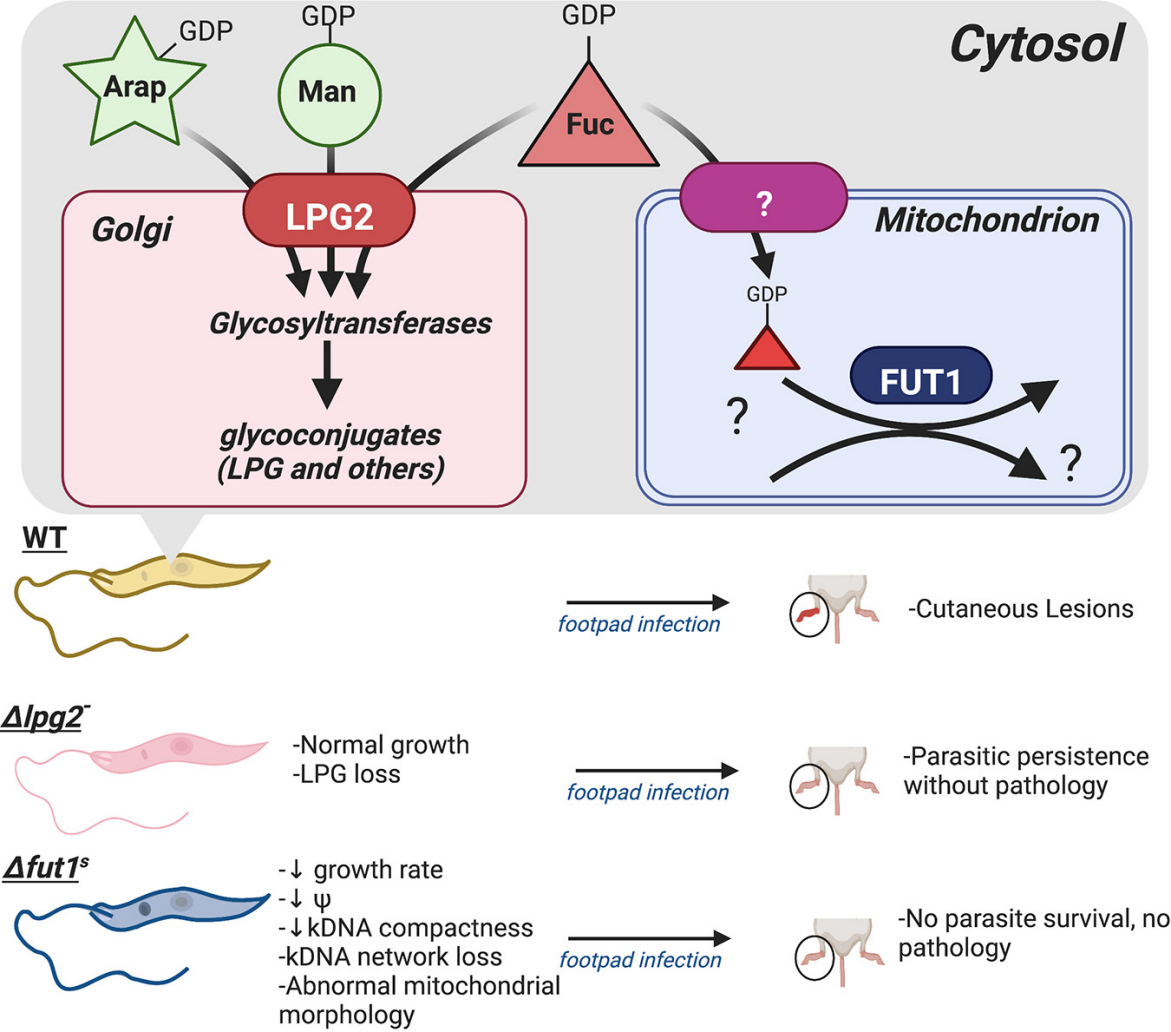
Overview of glycosylation pathways relevant to *LPG2* or *FUT1* biology and deletion mutants*. LPG2* encodes is a Golgi GDP-sugar transporter, essential for LPG synthesis. Mutant Δ*lpg2*^–^ parasites grow comparably to WT parasites in culture but do not produce pathology. *FUT1* encodes a fucosyltransferase located in the parasite mitochondria, whose substrates remain unknown. Null-mutant Δ*fut1*^s^ parasites exhibit severe defects, including significantly decreased growth rate, abnormal mitochondrial morphology, decreased mitochondrial membrane potential (Ψ), and loss of kDNA compactness or the kDNA network altogether. Arap, arabinopyranose (green star); Man, mannose (green circle); Fuc, fucose (red triangle); LPG, lipophosphoglycan. This figure was created with BioRender.com.

Leishmaniasis is a devastating parasitic disease caused by species of the trypanosomatid protozoan parasite genus *Leishmania*. Currently, there are over 12 million cases, with 1.7 billion people at risk of infection worldwide, and estimates of asymptomatic infections ranging as high as >300 million (1–4). Clinical manifestations include self-healing, localized or diffuse cutaneous lesions (cutaneous leishmaniasis), destruction of the nasopharyngeal mucosa (mucocutaneous leishmaniasis), and enlargement of the spleen or liver, which can lead to death (visceral leishmaniasis) (3). *Leishmania* parasites are transmitted to mammalian hosts via the bite of phlebotomine sand flies and undergo a series of transformations throughout their life cycle, predominantly as promastigotes (insect stage) and amastigotes (intracellular in mammalian host) (3, 5). Powerful genetic tools have uncovered a number of loci that are essential to the completion of the parasite’s life cycle and/or potentially suitable as targets for chemotherapy (6–10). Here, we focus on two mutants relevant to such efforts, whose properties are depicted in Fig. 1.

*Leishmania* parasites are completely coated in a dense glycocalyx largely containing glycoconjugate lipophosphoglycan (LPG), composed of (i) a 1-*O*-alkyl-2-lyso-phosohotidylinositol lipid anchor, (ii) a heptasaccharide core, (iii) a phosphoglycan polymer consisting of 15 to 30 Galβ1,4Manα1-PO_4_ repeating units (phosphoglycan [PG] repeats), often bearing side chain sugars such as galactose in Leishmania major, and (iv) a small oligosaccharide cap (11–13). Biochemical and genetic studies of L. major Δ*lpg1*^–^ (this nomenclature indicates homozygous deletion and precise replacement of the LPG1 ORF) mutants specifically lacking LPG have implicated it in many key steps of the parasite infectious cycle: in the sand fly vector, as well in the establishment of infection within the mammalian host following the sand fly bite (14–18). However, the amastigote stage lacks significant levels of LPG, and the few Δ*lpg1*^–^ parasites able to survive the initial host response are thereafter highly virulent (17). In contrast, deletion of *LPG2*, encoding a Golgi nucleotide sugar transporter (NST; described below), resulted in loss of LPG along with related glycoconjugates, including proteophosphoglycans, which are normally expressed in amastigotes (19). While many Δ*lpg2*^–^ phenotypes in both sand flies and establishment of mammalian infection mirrored those of Δ*lpg1*^–^ mutants, infections of susceptible mice with Δ*lpg2*^–^ mutants showed no overt disease and long-term persistence of a small number of parasites typical of long-term asymptomatic infections (19). This “persistence-without-pathology” phenotype induced protective immunity in a manner similar to that seen in natural healed infections (20).

Although the attenuated Δ*lpg2*^–^ phenotype had been attributed solely to the loss of LPG and related glycoconjugates (19), this view was challenged by studies of an analogous LPG-deficient mutant obtained by genetic deletion of the partially redundant *LPG5A* and *LPG5B* genes, encoding the Golgi UDP-galactose transporters (21). These parasites resemble the Δ*lpg2*^–^ mutant in lacking LPG and related PG-bearing glycoconjugates but, unlike Δ*lpg2*^–^ parasites, retained pathology following inoculation into susceptible mice, similar to that seen with Δ*lpg1*^–^ mutants lacking only LPG (22). This suggested the possibility of *LPG2*-dependent “off-LPG/phosphoglycan” effects, identification of which could shed new insight on both the persistence-without-pathology phenotype of the Δ*lpg2*^–^ strain, its ability to serve as a live vaccine line, and its tendency to revert toward virulence via second-site events (20, 23, 24).

The second mutant studied arises from finding that LPG2 was the first example of a multispecific NST, able to transport GDP-l-fucose as well as GDP-d-arapyranose (25). While the roles of GDP-Man in phosphoglycan repeat synthesis and d-Arap as a “capping” sugar able to block LPG interaction with the sand fly epithelium had been thoroughly studied (26, 27), GDP-Fuc transport was enigmatic. Although L. major expresses low levels of GDP-fucose mediated by the closely related bifunctional salvage enzymes AFKP80 and FKP40 (28, 29), convincing evidence for fucoconjugates has been hard to find. However, deletion of the two salvage enzymes could not be achieved unless “metabolic complementation” of GDP-fucose was engineered through expression of the trypanosome *de novo* GDP-fucose-synthetic pathway (29). Reasoning that this implied the existence of an essential fucosyltransferase (FUT), we identified 5 candidate fucosyltransferases, four of which appeared to be targeted as expected to the parasite secretory pathway. *SCA1* and *SCA2* function in d-Arap modifications of LPG (26), while null mutants of *SCAL* and *FUT2* showed little phenotype in culture (30).

The fifth candidate, *FUT1*, however, was important for parasite survival and potentially the key player in the GDP-fucose requirement. Unlike the other candidates, *FUT1* was found throughout all trypanosomatid species but not in other organisms (30, 31). Unexpectedly, FUT1 was found to target the parasite mitochondrion, as does its homolog TbFUT1 from Trypanosoma brucei, and both the mitochondrial localization and fucosyltransferase activity were essential (30, 31). While the gene was impossible to knock out by conventional approaches, through plasmid shuffling and examination of more than 1,000 events, a single, rare *Leishmania* Δ*fut1* deletion segregant (Δ*fut1*^s^ mutant) was obtained (30). This mutant line displayed severe growth and mitochondrial defects, which were rescued by L. major *FUT1* (Lmj*FUT1*) or Tb*FUT1* re-expression (30). While FUT1 proteins described in other organisms are able to fucosylate a variety of glycan substrates (32, 33), recombinant L. major FUT1 was unexpectedly able to fucosylate both glycan and peptide substrates *in vitro* (30). Thus far, neither the native acceptor in *Leishmania* nor that in trypanosomes has been identified, despite considerable effort in both species (30, 31).

Here, we generated high-coverage proteomes of wild-type (WT), Δ*lpg2*^–^, and Δ*fut1*^s^ Leishmania major, identifying over 6,500 proteins representing nearly 80% of the predicted proteome, expanding the previously known experimental L. major proteome by nearly 2-fold. Differential proteomic analysis showed numerous differences in the Δ*lpg2*^–^ and Δ*fut1*^s^ parasites, including some impacted in both. Despite the depth of this analysis, we failed to identify any fucose-bearing proteins across these proteomes. Furthermore, while glycopeptide enrichment analysis of WT samples also identified numerous instances of N-linked glycopeptides, no O-fucosylation or fucosylated N-linked glycans were observed. Taken together, this work highlights the challenges of identifying FUT1 targets and indicates that alternative approaches will be required in future studies.

## RESULTS

### Acquisition of a high coverage of the L. major proteome.

We examined the WT Fn strain of L. major, whose genome is one of the best-characterized references for *Leishmania* spp., as well as L. major Fn Δ*fut1*^s^ (30) and a newly created Fn Δ*lpg2*^–^ mutant. CRISPR/Cas9 mutagenesis was used to readily generate homozygous open reading frame (ORF) replacements (see Fig. S1 in the supplemental material), and one Fn Δ*lpg2*^–^ clonal line (c14.2) was chosen for further study. Preliminary studies showed that it lacks LPG by agglutination tests and does not exhibit lesion pathology when inoculated into susceptible mice but persists at low levels, similar to the previously described *L major* LV39 clone 5 Δ*lpg2*^–^ mutant (19). Previous studies showed that restoration of *LPG2* expression restores all WT phenotypes tested in both the Fn and LV39 clone 5 backgrounds (19, 34).

For each line, four replicate cultures were initiated and harvested in logarithmic growth phase. Parasite lysates were digested with trypsin and separated into 12 concatenated fractions by basic reverse-phase C_18_ chromatography (35) and then individually separated and analyzed by liquid chromatography-tandem mass spectrometry (LC-MS/MS). To ensure the ability to localize any potential glycosylation events, precursors were subjected to both higher-energy collisional dissociation (HCD) and electron transfer dissociation (ETD) fragmentation with spectra searched against a database consisting of the predicted L. major proteome. Protein matches meeting a 0.01 false discovery rate (FDR) cutoff, excluding contaminants, reverse decoys, and those identified only by site, were retained for an initial analysis of the coverage (Table S1).

**FIG 2:**
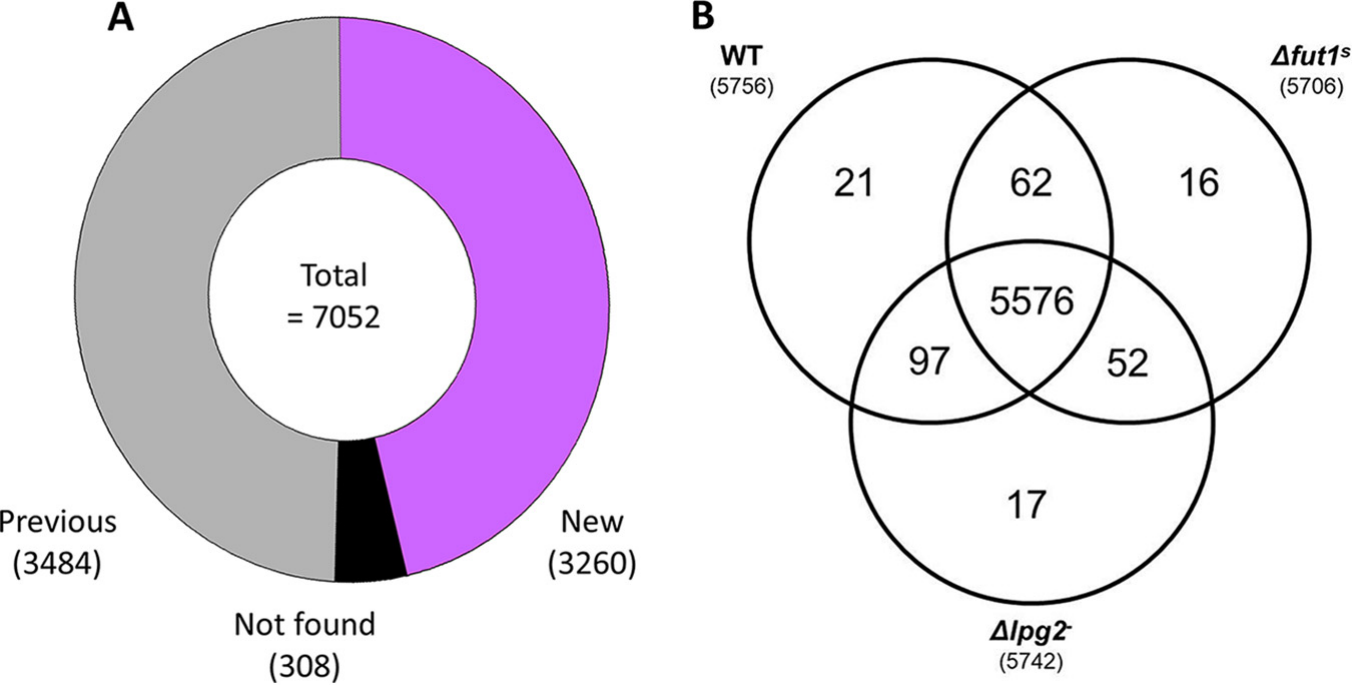
(A) Comparison of proteins identified in this work with previous studies. The combined data set (Table S1) from all lines and replicas was compared to L. major proteins with MS-based evidence annotated in TriTrypDB (https://tritrypdb.org/tritrypdb/app). Of the proteins identified here, 3,484 showed previous MS evidence and 3,260 had no prior MS-based reports; 308 proteins with previous MS-based evidence did not appear in our data sets. (B) Distribution of identified proteins among WT, Δ*fut1*^s^, and Δ*lpg2*^–^ L. major. The total proteome (Table S1) was further parsed by considering only proteins identified in at least two of the four biological replicates, in one or more parasite lines, yielding a total of 5,841 (Table S3). The Venn diagram displays the overlap of proteins among the three lines.

The numbers of proteins detected in WT, Δ*lpg2*^–^, and Δ*fut1*^s^ strains were similar (6,208, 6,102, and 5,934, respectively), totaling 6,744 (Table S1) and representing nearly 80% of the predicted L. major proteome (8,307 and 8,038 in TriTrypDB and UniProt, respectively). In comparisons with two previous proteomic studies of L. major (36, 37), 3,484 proteins identified had no prior MS-based evidence, while 3,260 had prior MS-based evidence, and 308 detected previously did not appear in our data set (Fig. 2A). Thus, this work increased experimental coverage of the *Leishmania* proteome by nearly 2-fold.

No predicted mitochondrion-encoded proteins were identified, despite the inclusion of many of them in our predicted proteome database (described in Materials and Methods). Often these are omitted from proteomic computational analysis completely, and due to their hydrophobicity, they are often not detected by standard or general approaches similar to those used here (38–40).

### Protein glycosylation and absence of detectable fucosylation.

We showed previously that recombinant FUT1 is able to modify both glycan and peptide substrates *in vitro* (30), and the expanded proteome provided an opportunity to search for evidence of *in vivo* fucosylation. To allow the localization of fucosylation events, ETD MS fragmentation was used, as O-fucosylated peptides are poorly localized with collision-based approaches (41). We searched first for peptide-*O*-fucose by considering deoxyhexose (dHex) modifications of serine or threonine residues, yet despite our proteome depth and the ability of our group to identify glycosylation events from deep proteome data sets (42, 43), no high-confidence O-fucosylation events were observed. Moreover, we were specifically unable to detect HSP70 or HSP60 peptide *O*-fucose, inferred previously in Leishmania donovani (44), peptides of which were fucosylated by recombinant FUT1 *in vitro* (30). Open searching (45) was also undertaken using MSFragger (46), which also failed to reveal any putative fucose-containing glycopeptides from our deep proteome analysis.

To further explore the diversity of glycans which may exist within L. major, we surveyed glycopeptides derived from L. major using zwitterionic-hydrophilic interaction liquid chromatography (ZIC-HILIC) (47) coupled to LC-MS analysis. Using open searching with MSFragger (46), multiple unique glycoforms were observed, the majority of which corresponded to N-linked glycoforms. While short O-linked glycopeptides can be enriched with this approach (48), our observation of predominately N-linked glycopeptide supports the idea that these escaped detection in L. major using ZIC-HILIC-based enrichment. We identified 65 glycopeptides bearing a variety of high-mannose N-linked glycans, as reported previously for *Leishmania* (49–52). The glycopeptides mapped to 49 different proteins, with 14 showing multiple sites and/or glyco-heterogeneity; 24 were predicted as hypothetical proteins of unknown function. The annotated glycosylated proteins are summarized in Table 1 and include many described previously, such as gp63/leishmanialysin, nucleotidases, and phosphatases (50), or ones likely to be so due to the presence of targeting motifs for the secretory pathway such as *FUT2* (30). While these findings highlight the idea that multiple N-linked glycopeptides could be recovered from L. major, no fucosylated N-linked glycans were observed (Table S2). Within this data set, three additional proteins absent from the total proteome were identified, bringing the collective total identified to 6,747. These three were all hypothetical proteins, two without prior MS-based evidence (LmjF.08.0350 and LmjF.09.1330) and one previously reported (LmjF.33.1035) (36). Both LmjF.08.0350 and LmjF.09.1330 have a detected mass shift of approximately 1,380.5 Da, while LmjF.33.1035 presented a mass shift of 2,803.3 Da. Future work will be required to confirm these preliminary assignments and the potential identity of the attached modifications.

**TABLE 1:** First-pass L. major glycoproteome from MS analysis of HILIC-enriched glycopeptides^*a*^

Protein annotation	ID
ATP pyrophosphate-lyase	LmjF.17.0200
β-Galactofuranosyl transferase (*LPG1*)	LmjF.25.0010
C_8_ sterol isomerase-like protein	LmjF.29.2140
Cathepsin L-like protease	LmjF.08.1060
Chitinase	LmjF.16.0790
d-Alanyl-glycyl endopeptidase-like protein	LmjF.33.2850
Dolichyl-diphosphooligosaccharide–protein glycotransferase	LmjF.35.1130
GPI ethanolamine phosphate transferase 3	LmjF.24.0340
Hypothetical predicted multipass transmembrane protein	LmjF.24.0700
Leishmanolysin (GP63)	LmjF.10.0465
Phosphoglycan β1,3-galactosyltransferase 3 (*SCG3*)	LmjF.02.0010
Putative 3′-nucleotidase/nuclease	LmjF.12.0400
Putative 3′-nucleotidase/nuclease	LmjF.31.2310
Putative amastin-like surface protein	LmjF.34.1080
Putative β-fructofuranosidase	LmjF.04.0310
Putative DNAJ domain protein	LmjF.24.0520
Putative extracellular receptor	LmjF.19.0640
Putative glycosyl transferase (*FUT2*)	LmjF.02.0330
Putative phospholipid:diacylglycerol acyltransferase	LmjF.09.1040
Putative surface protein amastin	LmjF.30.0860
Signal recognition particle receptor subunit β	LmjF.33.2620
Signal sequence receptor subunit α	LmjF.22.0260
Stealth CR3 domain-containing protein	LmjF.16.1010
Transmembrane 9 superfamily member	LmjF.34.3660
Uncharacterized protein L7845.03 (thioredoxin)	LmjF.35.1330

a Annotated proteins identified in the glycoproteomic analysis in this work. Gene IDs and protein annotations are taken from TriTrypDB (www.tritrypdb.org). Another 24 hypothetical proteins lacking annotation were also identified. GPI, glycosylphosphatidylinositol.

### WT and mutant cell lines display qualitatively similar proteomes.

To compare the impacts of *LPG2* and *FUT1* loss, we first created a high-confidence data set by parsing the total proteome (Table S1) for proteins detected in two or more biological replicates, in at least one cell line. This retained 5,841 proteins (Table S3), of which 95% (5,576) occurred in all three lines (Fig. 2B), evidence of high congruency. Of the remainder, 16 to 21 were unique to one line, and 62 to 97 were shared by two lines (Fig. 2B). As expected, the LPG2 protein was detected in all WT and Δ*fut1*^s^ lysates but not in Δ*lpg2*^–^ replicas. FUT1 peptides, on the other hand, were detected in only one WT biological replicate, perhaps due to low abundance (53).

### Mitochondrial proteome and gene ontology.

**FIG 3:**
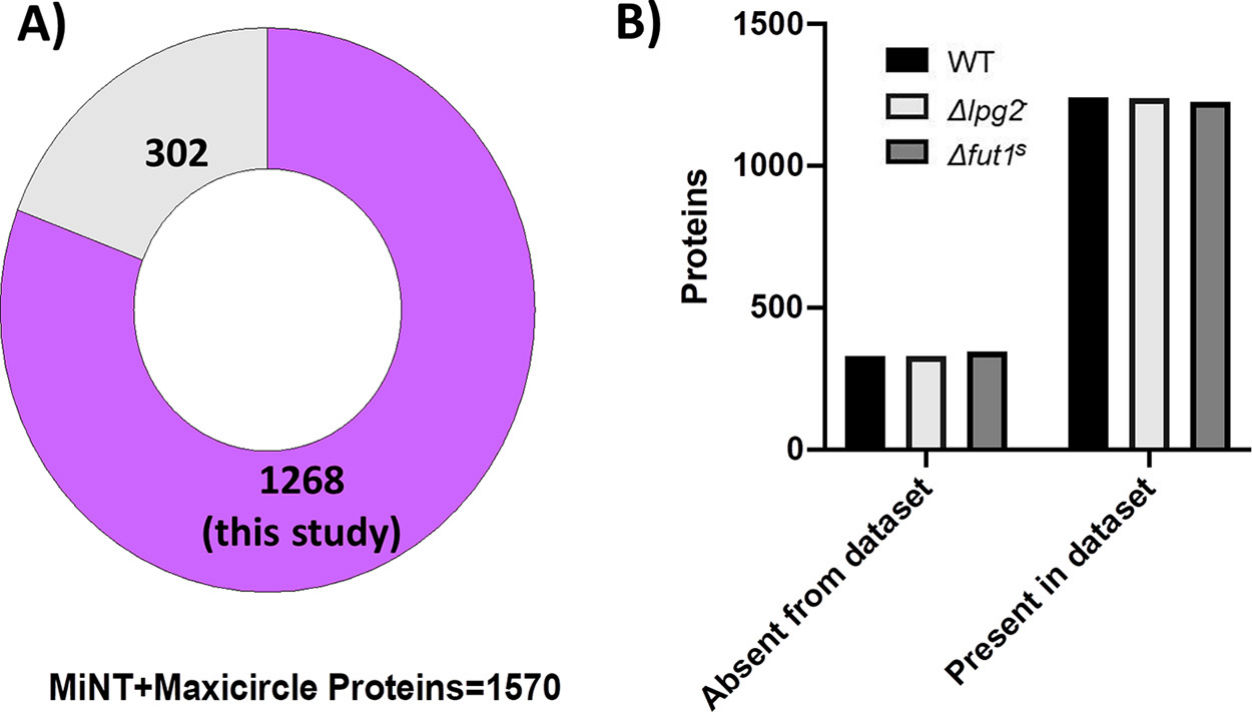
Representation of the predicted L. major mitochondrial proteome. Proteins from WT or mutant parasite lines (Table S3) were compared to proteins in MiNT (predicted nuclearly encoded mitochondrial protein), supplemented with maxicircle (mitochondrion)-encoded proteins as described in Materials and Methods. (A) A total of 1,268 proteins, representing 81% of the mitochondrial proteome, were identified in this study. (B) Distribution of mitochondrial proteins by cell line.

As FUT1 is targeted to the mitochondrion, where its loss results in numerous mitochondrial abnormalities (30), we queried the high-confidence proteomic data set for nuclearly encoded mitochondrial proteins present in the trypanosomatid predicted mitochondrial protein database MiNT (54). Of 1,559 mitochondrial L. major proteins in the MiNT database (approximately 19% of the total proteome), 1,268 (81%) were identified (Fig. 3A; Table S3), with the WT having a slightly higher number of identified proteins (1,241), followed by the Δ*lpg2*^–^ (1,239) and Δ*fut1*^s^ (1,226) mutants (Fig. 3B). Quantitative changes were apparent (discussed below), and no mitochondrially (maxicircle) encoded proteins were identified, as discussed above.

A gene ontology (GO) analysis was performed using the PANTHER classification system (Fig. S2; Table S4) (55). As seen previously (36), much of the L. major proteome consists of hypothetical proteins lacking annotation (over 60%) (Fig. S2A), and GO analysis of annotated proteins showed considerable similarity among all three lines. For biological processes (Fig. S2B), the categories with the highest number of genes were cellular process (GO:0009987), metabolic process (GO:0008152), localization (GO:0051179), and biological regulation (GO:0065007). For cellular components (Fig. S2C), the most commonly assigned categories were cellular anatomical entity (GO:0110165), intracellular (GO:0005622) and protein-containing complex (GO:0032991). For molecular functions (Fig. S2D), the categories were catalytic activity (GO:0003824) and binding (GO:0005488).

### Knockouts of *FUT1* and *LPG2* have significant differential impacts on L. major protein abundance.

**FIG 4:**
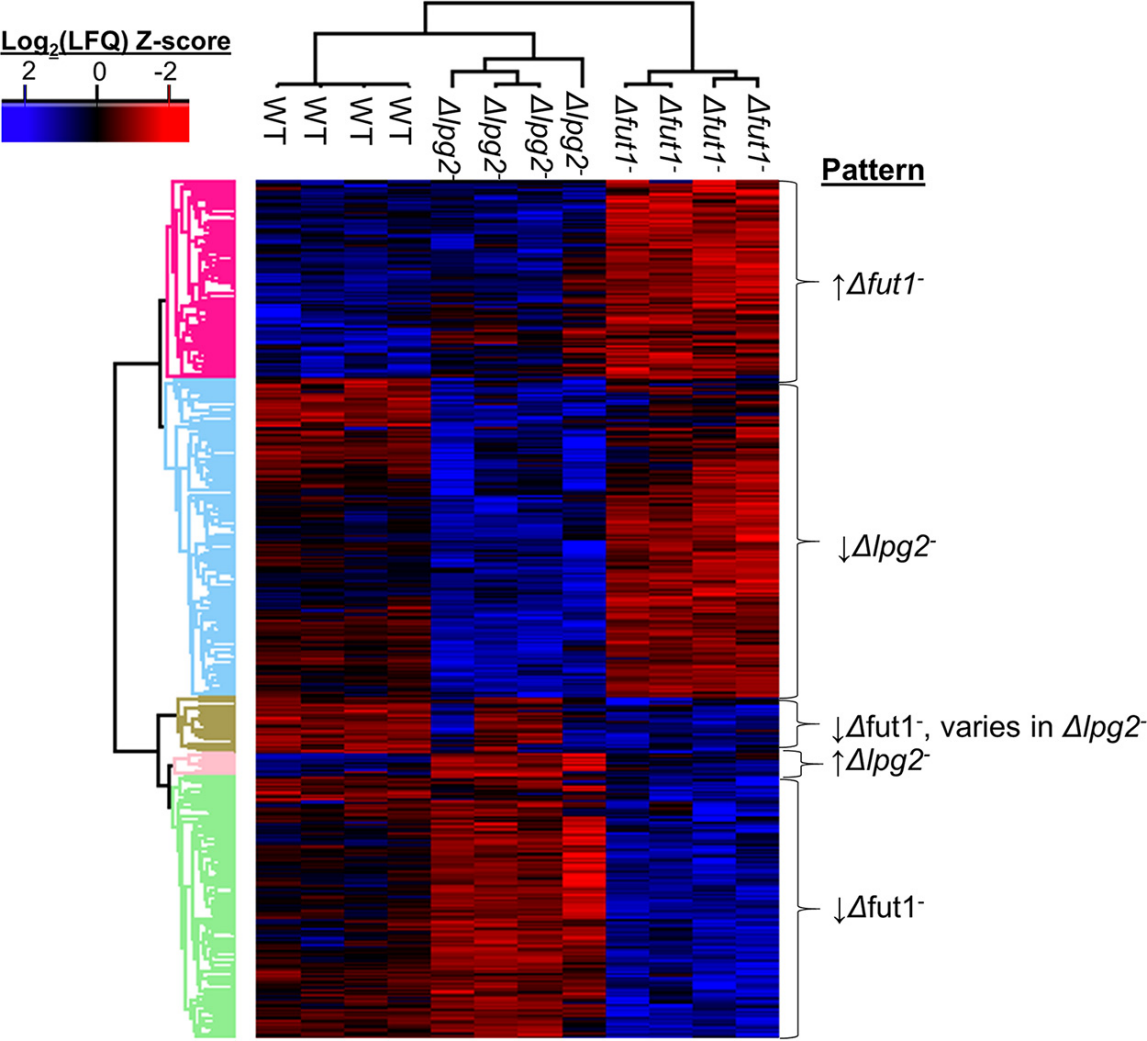
Patterns of variation of significantly differentially expressed proteins among WT and *FUT1* and *LPG2* knockout L. major lines. ANOVA using an FDR of 0.05 and an S0 of 1 was performed on LFQ values for the high-confidence proteome (Table S3), to identify proteins that are significantly different between WT, Δ*fut1*^s^, and Δ*lpg2*^–^ clones. From these data, a heat map was generated, clustering the parasite lines, each with four replicas (top dendrogram), and the differentially expressed genes (left dendrogram). The cluster properties are summarized on the right, and the specific proteins included in each cluster can be found in Table S5 and S6.

Quantitative differences among WT, Δ*fut1*^s^, and Δ*lpg2*^–^ parasites were assessed by analysis of variance (ANOVA) with an FDR of 0.05 applied to the protein abundance values (label-free quantitation [LFQ]) (Table S3), which were then normalized by Z-scoring to construct a heat map (Fig. 4). Clustering showed clear grouping of replicates within each line, as well as blocks encompassing 465 proteins differing significantly (Fig. 4). Of these, 107 increased and 142 decreased in Δ*fut1*^s^ parasites only, 173 decreased and 13 increased in Δ*lpg2*^–^ parasites only, and 30 decreased in Δ*fut1*^s^ parasites but increased in 2 of 4 Δ*lpg2*^–^ biological replicates (Tables S3, S5, and S6; Fig. 4). Again, the majority of differentially expressed proteins were hypothetical proteins (254/465 total) (Table S3).

SHERP, a stationary-phase/metacyclic parasite marker (56), was not seen in any Δ*lpg2*^–^ replicates but was unexpectedly detected in log-phase WT and Δ*fut1*^s^ parasites at similar levels (Tables S1 and S3). These results were corroborated by Western blot analysis with anti-SHERP antisera (Fig. S3).

### Significantly different proteins accompanying the severe mitochondrial dysfunction of Δ*fut1*^s^.

**FIG 5:**
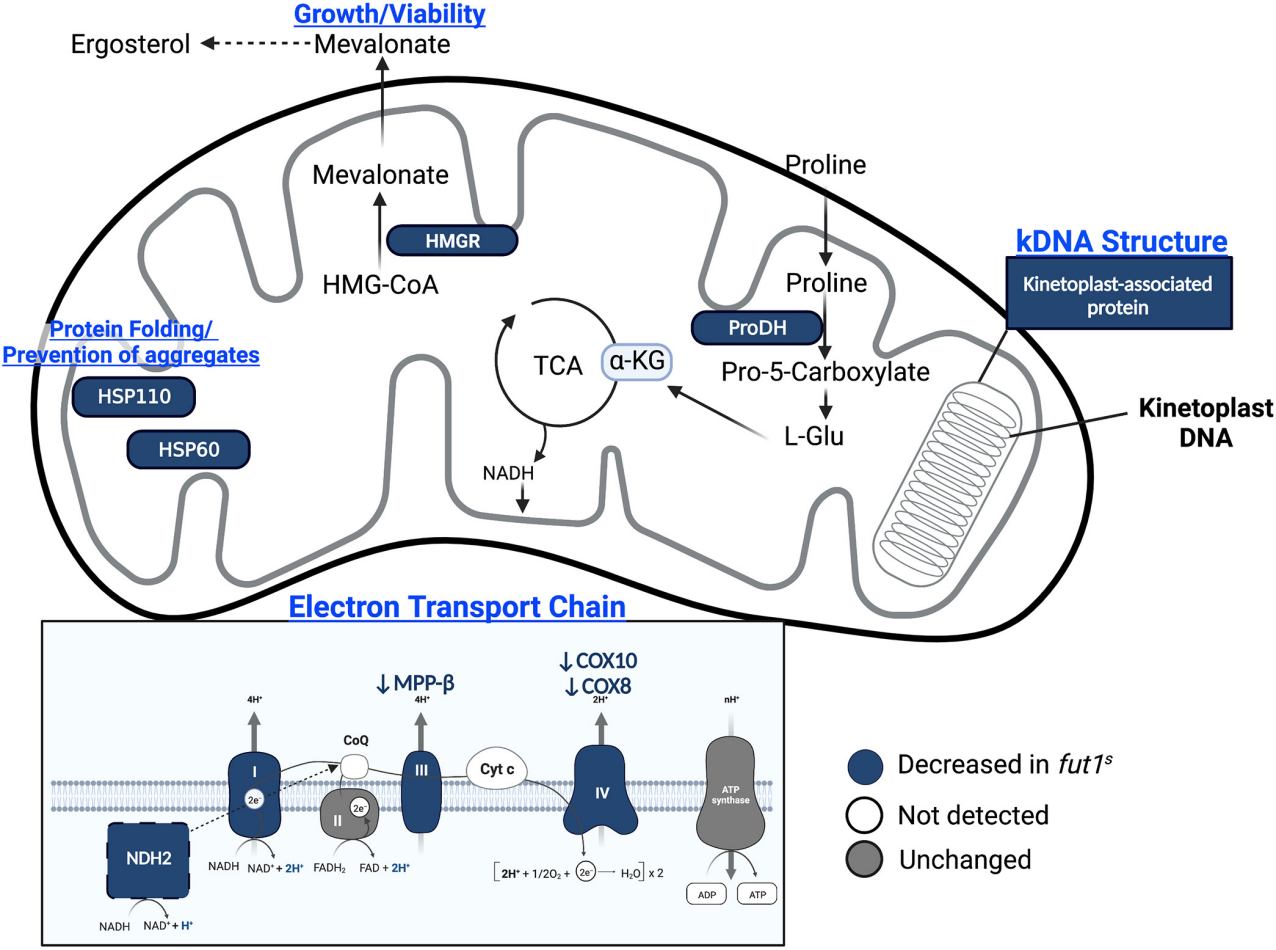
Schematic overview of a *Leishmania* mitochondrion showing proteins with significantly altered expression in Δ*fut1*^s^ relative to WT L. major. The specific proteins are listed in Table 1. Only mitochondrial proteins showing differential expression (Table 2) are shown. In the panel depicting the electron transport chain, the proteins decreased in the Δ*fut1*^s^ parasites are shown in blue, those not detected in our data set are shown in white, and those that were unchanged are shown in gray. It should be noted that trypanosomatids possess a single mitochondrion per cell whose structure differs from the typical metazoan mitochondrion shape depicted here. This figure was created with BioRender.com.

The Δ*fut1*^s^ mutant shows considerably slower growth and numerous mitochondrial abnormalities, including lowered mitochondrial membrane potential and complete loss or loss in compactness of the kinetoplast DNA (kDNA) network (30). From the cluster of 142 significantly less abundant proteins in Δ*fut1*^s^ parasites (Fig. 4; Table S5), 26 (18%) were predicted as mitochondrial, of which 13 were annotated (Table 2). Of 107 proteins that were significantly more abundant in Δ*fut1*^s^ parasites, 15 were mitochondrial, of which 2 were annotated (Table 2). The differentially expressed mitochondrial proteins are depicted in Fig. 5, many of which are positioned to contribute to Δ*fut1*^s^ mitochondrial dysfunction (see Discussion).

**TABLE 2:** Mitochondrial proteins significantly changed in Δ*fut1*^s^ parasites^*a*^

Cluster pattern	Protein name	TriTrypDB ID	Difference in log_2_(LFQ intensity)			Corrected ANOVA *P* value
			Δ*lpg2*^–^ vs WT	Δ*fut1*^s^ vs WT	Δ*fut1*^s^ vs Δ*lpg2*^–^	
Down in Δ*fut1*^s^	3-HMG-CoA reductase) (EC 1.1.1.34)	LmjF.30.3190	0.87	−1.38	−2.26	0.01
	Aldehyde dehydrogenase, mitochondrial (EC 1.2.1.3)	LmjF.25.1120	0.12	−2.00	−2.13	0.01
	Chaperonin HSP60, mitochondrial	LmjF.36.2030	0.80	−0.81	−1.61	0.04
	Kinetoplast-associated protein-like protein	LmjF.27.0240	1.60	−3.80	−5.40	0.00
	Metallopeptidase, clan ME, family M16 (EC 1.10.2.2)	LmjF.35.1380	0.26	−1.14	−1.40	0.04
	Proline dehydrogenase (EC 1.5.5.2)	LmjF.26.1610	1.34	−0.07	−1.40	0.02
	Putative asparaginyl-tRNA synthetase (EC 6.1.1.22)	LmjF.34.2340	0.42	−1.06	−1.48	0.03
	Putative carbamoyl-phosphate synthase (EC 6.3.4.16)	LmjF.16.0590	0.56	−0.69	−1.25	0.05
	Putative cytochrome *c* oxidase VIII (EC 1.9.3.1)	LmjF.31.1570	0.63	−2.16	−2.79	0.02
	Putative cytochrome *c* oxidase subunit 10 (EC 1.9.3.1)	LmjF.23.0370	−1.34	−2.49	−1.15	0.04
	Putative DNA-directed RNA polymerase I largest subunit (EC 2.7.7.6)	LmjF.32.0650	0.72	−0.69	−1.42	0.04
	Putative heat shock protein	LmjF.18.1370	0.56	−0.83	−1.38	0.04
	Putative NADH dehydrogenase (EC 1.6.99.3)	LmjF.36.5380	0.47	−1.20	−1.67	0.04
	Putative translation initiation factor	LmjF.17.1290	0.78	−0.59	−1.36	0.05

Up in Δ*fut1*^s^	Inositol phosphosphingolipid phospholipase C like	LmjF.08.0200	0.09	2.00	1.91	0.01
	Putative 3,2-*trans*-enoyl-CoA isomerase mitochondrial (EC 1.1.1.35) (EC 5.3.3.8)	LmjF.31.2330	−0.79	2.14	2.93	0.04

a Annotated mitochondrial proteins differing significantly in abundance, as determined by ANOVA, between the Δ*fut1*^s^ and WT or Δ*lpg2*^–^ parasites (Fig. 4; Tables S3 and S5). Overall, there were 279 proteins in clusters where proteins decreased in Δ*fut1*^s^ parasites (142 total; 26 mitochondrial, of which 13 are unannotated), increased in Δ*fut1*^s^ parasites (107 total; 17 mitochondrial, of which 15 are unannotated), or decreased in Δ*fut1*^s^ and varied in Δ*lpg2*^–^ parasites (30 total; 13 mitochondrial, of which 12 are unannotated). CoA, coenzyme A.

### Protein changes seen in the secretory pathway mutant Δ*lpg2*.

Overall, 174 proteins were significantly decreased in Δ*lpg2*^–^ parasites, of which 89 are of unknown function, while 13 increased in Δ*lpg2*^–^ parasites, of which 6 are of unknown function (Fig. 4; Tables S3 and S6). The predominance of proteins downregulated in Δ*lpg2*^–^ relative to Δ*fut1*^s^ organisms could be related to the relative health of the Δ*lpg2*^–^ mutant (this work and reference 19), as many of the changes seen in Δ*fut1*^s^ parasites involved stress responses arising from mitochondrial dysfunction. Notably, the proteins altered in Δ*lpg2*^–^ parasites mapped to a variety of cellular compartments and metabolic pathways (Tables S4 and S6), suggesting that despite the normal growth of this mutant in culture, the impact of loss of *LPG2* was nonetheless profound. The broad impact of *LPG2*-dependent effects thus hinders efforts to pinpoint those most important to the biological alterations of most interest, such as persistence without pathology.

*LPG2* loss affected several proteins involved in the glycosylation pathways. Two examples are decreases of phosphomannomutase (PMM) and phosphoacetylglucosamine mutase (PAGM), which catalyze the reversible transfer of phosphate between C-6 and C-1 hydroxyl groups of mannose and *N*-acetylglucosamine (57). PMM in particular is necessary for establishing infection in macrophages (27).

### Potential interactions between *LPG2-* and *FUT1*-dependent pathways.

While the proteins differentially altered in Δ*lpg2*^–^ and Δ*fut1*^s^ parasites were mostly quite distinct (Fig. 4; Tables S5 and S6), in 2 of 4 replicate Δ*lpg2*^–^ lines a small group of 30 proteins showed inverse regulation to changes observed in Δ*fut1*^s^ parasites (Fig. 4; Table S5). Of these, 12 were unannotated predicted mitochondrial proteins. As the replicates were grown at the same time from the same inoculation under the same conditions, we cannot account for the variability. Changes in Δ*fut1*^s^ parasites were also observed in proteins outside the mitochondrion (Table S5). Interestingly, these included increase of other glycosyltransferases, including α-1,2-mannosyltransferase, phosphoglycan β1,2-arabinosyltransferase, and phosphoglycan β1,3-galactosyltransferase 3 (Table S5). We speculate that the cross-mutant effects arise from competition for GDP-fucose synthesized in the cytosol (28, 29), for transport and use by secretory pathway fucosyltransferases (dependent on LPG2) or the mitochondrion (FUT1).

## DISCUSSION

This work presents a high-coverage L. major proteomic data set consisting of new mass spectrometry-based evidence for nearly 3,500 proteins beyond the 3,600 proteins typically identified experimentally in several *Leishmania* species, including L. major, by others (36). For quantitative analysis, the data were parsed to yield a high-confidence data set of 5,841 proteins. Comparison showed that the proteomes of WT, Δ*lpg2*^–^, and Δ*fut1*^s^ parasites were highly congruent, but with significant quantitative variation (58). Over half of the significantly changing proteins were uncharacterized hypothetical proteins.

### FUT1 and mitochondrial dysfunction.

In addition to severely delayed growth, the Δ*fut1*^s^ mutant exhibits profound mitochondrial dysfunction, including loss of membrane potential, bloated cristae, presence of large aggregates, loss of kDNA compactness, and complete loss of the kDNA network in some parasites (30). Benefiting from the high coverage of mitochondrial proteins in our data set (>80%) (Fig. 3), we were able to survey the impact of *FUT1* deletion on these (Table 2; Table S5; Fig. 4). The Δ*fut1*^s^ mutant had significantly decreased levels of key components of mitochondrial respiratory chain complexes III (MMP-β) and IV (cytochrome *c* oxidases COX8 and COX10), (59) as well as the alternative NADH dehydrogenase (NDH2) (58, 60). Δ*fut1*^s^ alterations in several maxicircle (mitochondrion)-encoded components of the respiratory chain could not be assessed, as they were absent in our data sets. Collectively, downregulation of this pathway matches the changes in membrane potential and growth seen in Δ*fut1*^s^ parasites (30).

Δ*fut1*^s^ parasites showed downregulation of kinetoplast-associated proteins (KAPs), histone-like proteins attributed to packaging mitochondrial DNA (kDNA) in trypanosomatids (61). Downregulation of these has been associated with rearrangement of the kinetoplast structure, parasite growth defects, and shrinkage and complete loss of the kDNA network (62, 63), all phenotypes seen in Δ*fut1*^s^ parasites (30).

Enzymes impacted in Δ*fut1*^s^ parasites include 3-hydroxy-3-methylglutaryl coenzyme A reductase (HMGR) and proline dehydrogenase (ProDH) (Table 2; Table S5; Fig. 5). HMGR is a key enzyme in the mevalonate pathway, yielding isoprenoids important for viability and proliferation (64). ProDH mediates the first step in a pathway that leads to production of α-ketoglutarate entering the tricarboxylic acid cycle (65) and generates FADH_2_, which transfers electrons to coenzyme Q and the electron transport pathways (65). ProDH transcripts have been found to be present in higher levels in drug-resistant *Leishmania* (66).

Several proteins associated with stress responses were also impacted in Δ*fut1*^s^ parasites, including mitochondrial heat shock proteins HSP60 and HSP110 (67). HSP60 acts as one of the principal mediators of protein homeostasis in the mitochondrial matrix and in response to damaged or misfolded mitochondrial proteins after oxidative stress (67, 68). The inositol phosphosphingolipid phospholipase C-like (ISCL) protein, which contributes to parasite survival in acidic environments, was increased in Δ*fut1*^s^ parasites (69).

### A first-pass L. major glycoproteome, lacking detectable fucosylation.

The FUT1 protein shows motifs characteristic of fucosyltransferases within the GT11 family, and recombinant Trypanosoma brucei and L. major FUT1 proteins were able to fucosylate various glycans *in vitro* (30, 31). Moreover, LmjFUT1 was shown to fucosylate several peptides *in vitro* (30 [TbrFUT1 was not tested]). The *in vivo* substrate of FUT1 has proven elusive in both parasite species, and the broad specificity of LmjFUT1 prompted us to search for protein O-fucosylation as part of this work. While glycopeptides were not detected with high confidence in the total proteomic studies, potentially reflecting technical considerations (70, 71), following HILIC enrichment we were able to identify 58 glycopeptides representing 49 *Leishmania* proteins, many of which were known or anticipated to be glycosylated but many of which were novel (Table 1). Several other potential modifications were identified computationally, such as pyrophosphorylation (72), which will require experimental verification. In Trypanosoma cruzi, HILIC enrichment similarly successfully identified a variety of N- and O-glycosylated proteins (73). Comprehensive studies of the *Leishmania* glycoproteome were lacking (50), with previous studies being mostly limited to a few glycoproteins such as GP63 (leishmanialysin). Despite the utility of the HILIC enrichment, many known glycosylated proteins of *Leishmania* were not detected, and thus, the proteins in Table 1 should be regarded as a first-pass (preliminary) glycoproteome, confirmation of which will be needed in any event.

Notably, we did not find evidence for peptide *O*-fucose or fucosylated glycans. Although glycopeptide enrichment using HILIC can be utilized for O-glycoproteome characterization, it may bias for peptides with large glycans or multiple glycosylations (74). It is possible that any potential *FUT1*-dependent protein fucosylation may be in a form recalcitrant to the methods used, or that other parasite stages could yield different results. It is also possible that further proteomic searches extending our ultradeep effort might eventually yield proteins or modifications (including fucosylation) not identified here. Alternatively, while it is difficult to prove a negative, the possibility that the *in vivo* substrate of FUT1 may be glycans or glycolipids rather than proteins or glycoproteins seems at least as likely from the absence of detectable peptide fucosylation. Resolution of this question ultimately will depend on identification of the relevant FUT1-dependent glycoconjugate in the future.

### Summary and perspective.

Proteins showing altered expression in Δ*lpg2*^–^ parasites could contribute to the persistence-without-pathology phenotype seen in this mutant; however, as these parasites grow normally in culture, we were challenged to compose a predictive query of the 186 differentially expressed candidates for those most likely to contribute to the phenotype. These data also raised the possibility that changes in the expression of one or more of these proteins could contribute to the recovery of amastigote virulence in Δ*lpg2*^–^ revertants occasionally found in infected animals (23). Experimental tests of the role(s) of the *LPG2*-dependent proteome in the loss or recovery of amastigote virulence will be required to resolve this. Similarly, Δ*fut1*^s^ parasites showed 249 differentially expressed proteins, including many known or potential mitochondrial proteins, as expected from its strong impact on mitochondrial function, as well as a number of nonmitochondrial proteins. While our studies raise many hypotheses for the roles of both FUT1 and LPG2 in parasite biology, genetic confirmation will be required, as well as studies to establish whether the effects seen are direct or indirect consequences of gene ablation. Fortunately, with the advent of high-throughput knockout screening via CRISPR technology (75), probing the importance of this panoply of genes will be increasingly feasible.

## MATERIALS AND METHODS

### Cell culture.

All parasites were derivatives of L. major strain Fn (MHOM/IL/80/Fn). Parasites were grown as the promastigote form *in vitro* in complete medium 199 (M199) supplemented with 10% heat-inactivated fetal bovine serum (FBS), 40 mM HEPES (pH 7.4), 100 nM adenine, 1 μg/mL biotin, 5 μg/mL hemin, penicillin-streptomycin, and 2 μg/mL biopterin. Parasites were seeded in 200 mL medium at a density of 10^5^/mL in roller bottles and allowed to grow until mid-log phase, corresponding to a density of 1 × 10^6^ to 4 × 10^6^/mL. Culture vessels were rotated at approximately 2 rpm using a cell production roller apparatus (Bellco Biotechnology). The Δ*fut1*^s^ segregant was described previously (30).

### Generation of a Δ*lpg2*^–^ mutant line by CRISPR/Cas9 mutagenesis.

Parasites were engineered to express a human codon-optimized Streptococcus pyogenes Cas9 and an LPG2 sgRNA (plasmid p63Phleo-HspCas9 or B7521; single guide RNA [sgRNA] synthesized from template B7617) expressed from a U6 promoter as described previously (76). To replace the *LPG2* ORF, homology-directed repair (HDR) templates were designed containing a hygromycin B (*HYG*) resistance gene flanked by sequences matching the 30 nucleotides flanking the *LPG2* ORF. A total of 10 μg of HDR DNA was transfected into the L. major HSpCas9-B7617 parasites, which were then plated on semisolid M199 medium containing 50 μg/mL hygromycin B. Numerous hygromycin B-resistant (HYG^r^) colonies were obtained, of which 26 were tested and appeared to be WT/Δ*lpg2*::*HYG* heterozygotes. Subsequently, parasites were transfected with a blasticidin (*BSD*) resistance gene HDR template, bearing the 30 nucleotides (nt) of *LPG2* flanking sequence as before. The HSpCas9-B7617-HYG^r^ parasites were transfected and plated onto semisolid medium containing 50 μg/mL hygromycin B and 10 μg/mL blasticidin. A total of 22 colonies were tested and confirmed to be Δ*lpg2*::*HYG*/Δ*lpg2*::*BSD* knockouts by PCR tests (Fig. S1), and preliminary data showed them to be LPG deficient, as judged by their failure to agglutinate with monoclonal anti-PG antibody WIC79.3. One clone was selected for further study (SpCas9-B7617 B+H-c14.2), referred to here as the Δ*lpg2*^–^ clone.

### Generation of parasite lysates for proteomic analysis.

Whole-parasite lysates were prepared as described previously (77) with some modifications. After 3 washes with ice-cold phosphate-buffered saline (PBS), parasites were resuspended in 1 mL of ice-cold lysis buffer [6 M guanidinium chloride, 100 mM Tris (pH 8.5), 10 mM Tris(2-carboxyethyl)phosphine (TCEP), 40 mM 2-chloroacetamide (CAA), supplemented with a protease inhibitor tablet (Roche) and 0.2 mg/mL 1,10-phenanthroline]. Samples were immediately boiled at 95°C in a ThermoMixer at 1,500 rpm. After cooling samples on ice for 10 min, an additional boiling step was done at 95°C. One milligram of protein was collected from four biological replicates. The samples were acetone precipitated with 4 volumes of ice-cold acetone at −20°C overnight and then a second time under the same conditions for at least 4 h. After centrifugation and supernatant removal, samples were air dried while covered with a Kimwipe and stored at −80°C until ready for analysis.

### Digestion of L. major proteome samples.

Precipitated protein pellets were resuspended in 50% trifluoroethanol and then heated at 50°C for 10 min with shaking at 1,000 rpm. Resuspended samples were then reduced/alkylated in a single step with 20 mM Tris(2-carboxyethyl)phosphine and 40 mM chloroacetamide for 1 h in the dark. Samples were then diluted 10-fold with 100 mM triethylammonium bicarbonate and digested with trypsin (1/100 [wt/wt]) overnight with shaking at 800 rpm. Digested samples were acidified to a final concentration of 0.5% formic acid and desalted with 50-mg tC_18_ Sep-Pak columns (Waters Corporation, Milford, MA, USA) according to the manufacturer’s instructions. Briefly, tC_18_ Sep-Pak columns were conditioned with buffer B (0.1% formic acid [FA], 80% acetonitrile [ACN]) and washed with 10 volumes of buffer A* (0.1% trifluoroacetic acid [TFA], 2% ACN), samples were loaded, columns were washed with 10 volumes of buffer A*, and bound peptides were eluted with buffer B and then dried by vacuum centrifugation.

### High-pH fractionation.

Proteome samples were fractionated by basic reverse-phase chromatography according to the protocol of Batth and Olsen (78) with minor modifications. Briefly, peptides were resuspended in 1 mL of buffer A (5 mM ammonium formate, pH 10.5) and separated using a 1100 series high-performance liquid chromatograph (HPLC) (Agilent Technologies, CA) using a Gemini NX C_18_ column (4.6 by 250 mm, 5 μm; Phenomenex, CA) at a flow rate of 1 mL/min. Separation was accomplished using a 90-min gradient with samples loaded on the column at 2% buffer B (5 mM ammonium formate, 90% ACN [pH 10.5]) for 3 min. The concentration of buffer B was then ramped from 2% to 28% over 45 min, from 28% to 40% over 5 min, and from 40% to 80% over 5 min. The gradient was held at 80% B for 2 min, and then the column was regenerated by being returned to 2% B over 10 min and held at 2% B for 10 min. Sixty 1-min fractions were collected. Every group of five fractions was combined to generate a total of 12 pooled fractions, which were concentrated by vacuum centrifugation, desalted using C_18_ stage tips, and then subjected to mass-spectrometric analysis.

### Proteomics analysis using reverse-phase LC-MS.

Pooled basic reverse-phase fractions were resuspended in buffer A* (2% ACN, 0.1% TFA) and separated using a two-column chromatography setup composed of a PepMap100 C_18_ 20-mm by 75-μm trap and a PepMap C_18_ 500-mm by 75-μm analytical column (Thermo Fisher Scientific). Samples were concentrated onto the trap column at 5 μL/min for 5 min and infused into an Orbitrap Elite mass spectrometer (Thermo Fisher Scientific). Then, 125-min gradients were run, altering the buffer composition from 2% buffer B (80% ACN, 0.1% FA) to 28% B over 95 min, from 28% B to 40% B over 10 min, and then from 40% B to 80% B over 5 min; the composition was held at 80% B for 3 min, decreased to 2% B over 2 min, and held at 2% B for another 10 min. The Elite Orbitrap mass spectrometer was operated in a data-dependent mode, automatically switching between the acquisition of a single Orbitrap MS scan (60,000 resolution) and a maximum of 10 MS-MS scans, with each ion subjected to both an HCD scan (15,000 resolution; normalized collision energy [NCE], 40; maximum fill time, 200 ms; automatic gain control [AGC], 5 × 10^4^) and an ETD scan (ion trap analyzed; ETD reaction time, 100 ms with supplementary activation enabled; AGC, 1 × 10^4^).

### Proteomic analysis.

Proteome samples were processed using MaxQuant (v1.6.3.4) (79) and searched against the Leishmania major MHOM (UniProt accession no. UP000000542, containing 8,038 proteins) and TriTrypDB Leishmania major Fn (TrypDB 37) databases. The reference proteome database was supplemented with predicted proteins from the kinetoplast maxicircle (mitochondrion) of L. major (LmjF00.0040, NADH dehydrogenase 7 [MURF3, ND7]; LmjF00.0050, cytochrome oxidase 3; LmjF00.0060, CYb; LmjF00.0070, MURF4 [A6]; LmjF00.0080, MURF1; LmjF00.0100, NADH dehydrogenase subunit 1 [ND1]; NADH dehydrogenase subunit 4, NADH dehydrogenase subunit 5, LmjF00.0110 cytochrome oxidase 2 [CO2]; LmjF00.0120, MURF2; LmjF00.0130, cytochrome oxidase 1 [CO1]; LmjF00.0150, NADH dehydrogenase 4 [ND4]; LmjF00.0180, NADH dehydrogenase 5 [ND5]); due to extensive pan-RNA editing, other protein products that could not be reliably predicted were not included. Searches were undertaken using trypsin enzyme specificity with carbamidomethylation of cysteine as a fixed modification. Oxidation of methionine and dHex modifications of serine/threonine residues were included as variable modifications, and a maximum of 2 missed cleavages was allowed. To attempt to identify any potential complex fucosylation events, dependent peptide searching was enabled. To ensure the inclusion of only high-quality peptide spectral matches (PSMs), a PSM FDR of 0.1% was set, while an FDR of 1% was allowed at the protein level. To enhance the identification of peptides between samples, the “match between runs” option was enabled, with a precursor match window set to 2 min and an alignment window of 20 min with the LFQ option enabled (80).

The result file was then uploaded to Perseus (V1.6.0.7) (81) for statistical analysis. Potential contaminants, proteins only identified by site, and reverse decoys were removed. For LFQ comparisons, values were log_2_(*x*) transformed and biological replicates were grouped. Data were parsed further by removing proteins not identified in at least two biological replicates in at least one cell line. Missing values were inferred based on the observed total peptide intensities with a range of 0.3σ and a downshift of 1.8σ using Perseus. A multiple-sample (ANOVA) test with a permutation-based FDR set at 0.05 and the artificial within groups parameter S0 set at 1 was performed to identify significantly differentially abundant proteins between Δ*fut1*^s^, WT, and Δ*lpg2*^–^ lines. A heat map of significantly different proteins was constructed after Z-score-based normalization and Euclidean clustering of the transformed LFQ values (82).

### Glycopeptide enrichment by ZIC-HILIC.

Two hundred fifty micrograms of whole-cell lysates that had been digested, C_18_ Sep-Pak cleaned, and dried was resuspended in 80% acetonitrile–1% TFA, and glycopeptides were enriched using homemade ZIC-HILIC stage tips as previously described (48). Briefly, ZIC-HILIC columns were first conditioned with 80% acetonitrile–1% TFA; then, samples were loaded onto columns before being washed with 80% acetonitrile–1% TFA, and glycopeptides were eluted with Milli-Q water. Samples were dried and stored at −20°C until undergoing LC-MS.

### LC-MS analysis of ZIC-HILIC-enriched samples.

ZIC-HILIC-enriched samples were resuspended in buffer A* and separated using a two-column chromatography setup composed of a PepMap100 C_18_ 20-mm by 75-μm trap and a PepMap C_18_ 500-mm by 75-μm analytical column (Thermo Fisher Scientific) coupled to an Orbitrap Fusion Lumos Tribrid mass spectrometer (Thermo Fisher Scientific). ZIC-HILIC-enriched samples were analyzed using 185-min gradients. Separation gradients were run for each sample, altering the buffer composition from 2% buffer B to 28% B over 106 or 166 min depending on the run length, from 28% B to 40% B over 9 min, and then from 40% B to 80% B over 3 min; the composition was held at 80% B for 2 min, dropped to 2% B over 2 min, and held at 2% B for another 3 min. The Lumos mass spectrometer was operated in a data-dependent mode with a single Orbitrap MS scan (350 to 1,800 *m/z*, maximal injection time of 50 ms, an AGC maximum of 4 × 10^5^ ions, and a resolution of 120,000) acquired every 3 s followed by Orbitrap MS/MS HCD scans of precursors (NCE, 30%; maximal injection time of 100 ms; AGC set to a maximum of 1.0 × 10^5^ ions; and a resolution of 15,000). HCD scans containing the oxonium ions (204.0867; 138.0545 and 366.1396 *m/z*) triggered two additional product-dependent MS/MS scans of potential glycopeptides; an Orbitrap electron transfer–higher-energy collision dissociation [EThcD] scan (NCE, 15%; maximal injection time of 250 ms; AGC set to a maximum of 2 × 10^5^ ions with a resolution of 30,000) and an ion trap collision-induced dissociation (CID) scan (NCE, 35%; maximal injection time of 40 ms; AGC set to a maximum of 5 × 10^4^ ions). Data files were searched using MSFragger (v15) (46) using the Leishmania major MHOM proteome (UniProt accession no. UP000000542). Open database searches were performed, allowing modifications between −150 and 1,000 Da on the deep proteome analysis, with global.modsummary.tsv used to assess the presence of fucosylated modifications. For the detection of glycoforms within ZIC-HILIC enrichments, open database searches were performed, allowing modifications between −150 and 2,000 Da. The results from the ZIC-HILIC open searches were combined within R, and only assignments with an MSFragger expectation value of <0.001 and a delta mass of >140 Da were retained for analysis.

### Data availability.

All MS data, search results, and R scripts have been deposited in the PRIDE ProteomeXchange Consortium repository (83, 84) with identifiers PXD015966 and PXD035738.
