# Supplementary material for: Expanded Proteomic Survey of the Human Parasite Leishmania major Focusing on Changes in Null Mutants of the Golgi GDP-Mannose/Fucose/Arabinopyranose Transporter LPG2 and of the Mitochondrial Fucosyltransferase FUT1
Source: Microbiol Spectr. 2022 Nov 17;10(6):e03052-22. doi: 10.1128/spectrum.03052-22 (PMC9769760; doi:10.1128/spectrum.03052-22)
Supplement: Supplemental file 5 — Supplemental material. Download spectrum.03052-22-s0005.pdf, PDF file, 0.4 MB [file spectrum.03052-22-s0005.pdf]

October 26, 2022

Supplementary information

Polanco, Scott, Lye and Beverley, "An expanded proteomic survey of the human parasite *Leishmania major* focusing on changes in null mutants of the Golgi GDP-Mannose/Fucose/Arabino*pyranose* transporter *LPG2* or the mitochondrial fucosyltransferase *FUT1*"

Contents: Legends for Supplementary Tables S1-S6

Supplementary Figures S1-S3

Supplementary Tables S5, S6.

Supplementary Tables

Please note that due to their size, Tables S1-S4 are included as on-line supplementary information.

Supplementary Table S1.

Total *L. major* proteome. All proteins identified by one or more peptide in WT, $\Delta lpg2^-$ or $\Delta fut1^s$ total or HILIC-enriched datasets were included._A summary comparing this dataset with previous work is found in Fig. 2A. The heading terms are defined below

- **Protein Names:** Names of proteins contained within group based on shared identified peptides
- **Gene names:** Names of genes associated with identified proteins
- **Protein IDs :** All proteins consistent with identification criteria. Matches were made to a database composed of TriTrypDB and UniProt identifiers.
- **Majority Protein IDs:** Protein(s) matching at least half the peptides matched to the protein group. These IDs were used for analyses and data interpretation.
- **TriTryp IDs**: Identifiers from TriTrypDB database associated with majority protein IDs
- **No Prior MS evidence listed:** Protein groups identified in this study, that had no MS-based evidence listed on TriTrypDB for *L. major*

- **Prior MS evidence listed:** Protein groups identified in this study, that had MS-based evidence listed on TriTrypDB for *L. major*
- **Retained for analysis of biological significance:** Proteins used for downstream analyses to determine biological significance of *LPG2* or *FUT1* deletion. Only proteins that were detected in at least two biological replicates within one or more parasite line were considered.
- **Log2 (LFQ intensity):** relative label-free quantitative value across all samples (LFQ intensity) after log2 transformation
- **Peptides:** Number of peptides associated with the protein(s)
- **Razor + Unique peptides:** Razor peptides are found in more than one protein group but assigned to the group with the highest number of identified peptides.
- **Unique peptides:** Peptides matching only one protein sequence within a group
- **Sequence coverage [%]:** Percent sequence coverage by identified peptides belonging to the best protein sequence in the group
- **Unique + razor sequence coverage [%]:** Coverage of protein sequence based on both unique and razor peptides identified belonging to the best protein sequence in the group
- **Mol. Weight [kDa]:** Molecular weight corresponding to the best protein in the group
- **Q-value:** False discovery rate within the protein group
- **Score:** Andromeda score measuring efficiency of matching theoretical fragment masses to the acquired spectra. The value is calculated as the $-\log_{10}$ (probability match is acquired by chance).
- **Intensity:** Sum of intensities for all peptides corresponding to the protein group
- **MS/MS count:** Number of spectra for the protein group

Supplementary Table S2.

Peptides and proteins identified by MS following HILIC enrichment. The first tab shows peptides, the second tab shows modified peptides collapsed by modification and/or protein; the third tab shows N-linked glycopeptides, the fourth tab shows N-linked glycoproteins, and the fifth tab shows pyrophosphorylation or unidentified modifications. A summary of those glycoproteins showing database annotations is found in Table 1.

Supplementary Table S3.

High confidence proteome of *L. major*. The total experimental proteome (Table S1) was parsed to retain only those proteins present in 2/4 replicas in one or more lines. Their representation amongst lines appears in Fig. 2B.

Supplementary Table S4 .

Gene ontology assignments. A summary of these appears in Fig S2.

Supplementary Table S5.

Annotated Proteins Significantly Affected in *Δfut1*^{s}. Proteins differing significantly in abundance between the *Δfut1*^{s}, *Δlpg2*$^{-}$, and WT parasite lines were evaluated by ANOVA; those proteins within clusters changing in *Δfut1*^{s} are shown (Fig. 4; Table S3). Overall, there were 279 proteins in clusters where proteins decreased in *Δfut1*^{s} (142 total of which 70 are of unknown function), increased in *Δfut1*^{s} (107 total of which 70 are of unknown function), or decreased in *Δfut1*^{s} and varied amongst replicas in *Δlpg2*- (30 total of which 19 are of unknown function). Proteins present in the MiNT database are indicated, as are the corresponding cellular component GO terms when available. A diagram of depicting the annotated mitochondrial proteins is shown in Fig. 5.

Supplementary Table S6.

Annotated Proteins Significantly Affected in *Δlpg2*$^{-}$. Proteins differing significantly in abundance between the *Δfut1*^{s}, *Δlpg2*$^{-}$, and WT parasite lines were evaluated by ANOVA; those proteins within clusters changing in *Δlpg2*$^{-}$ are shown (Fig. 4; Table S3). Overall, there were 174 proteins significantly decreased in *Δlpg2*$^{-}$, of which 89 are of unknown function, while 13 increased in *Δlpg2*$^{-}$ of which 6 are of unknown function. A third group of 30 proteins, of which 19 are of unknown function, were significantly decreased in *Δfut1*^{s} but varied among the biological replicates in *Δlpg2*$^{-}$ (Table S5).

Supplementary Figures

Supplementary Figure 1.

PCR confirmation of *L.major* Fn homozygous *LPG2* knockouts (*Δlpg2*⁻) obtained by CRISPR/Cas9 mutagenesis. Template DNAs used were WT and two different *Δlpg2*⁻ clonal lines; 'Neg' corresponds to no template DNA added. Clone P14 was taken for proteomics studies presented here.

The upper panel show amplification with the hygromycin B drug resistance marker (HYG^r) with primers SMB2891 (5'-GGAGGACCCGGGCCACCATGAAAAAGCCTGAACTCACCG and SMB2892 (5'-GAGGATCTAGACTATTCCTTTGCCCTCGGACGA. The middle panel shows amplification with the blastocidin resistance marker (BSD^r) with primers SMB7919 (5'-CCAACCGAAAGAATTGCATCAGCAACTGTC CCACCATGGCCAAGCCTTTGTC) and SMB7920 (5'-CCCTTCTACGACTGCGGCTAACAACGGTGATTAGCCCTCCCACACATAACCA). The lower panel shows amplification for the *LPG2* ORF with primers SMB7914 (5'-TCTGTCAGTAACTCGATCGGCC) and SMB7915 (5'-CGTCTTGCCGGTCTGCTGCATC).

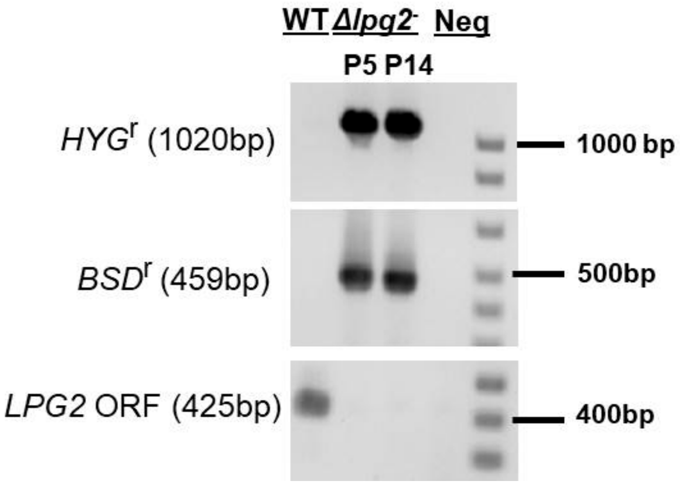

Supplementary Figure 2 .

Gene ontology (GO) analysis of the experimental *L. major* proteome. The gene ontology assignments were determined using the PANTHER classification system. A) Numbers of proteins able to be assigned or not. Classification of proteomic datasets from each cell line by B) biological process, C) molecular function, and D) cellular component.

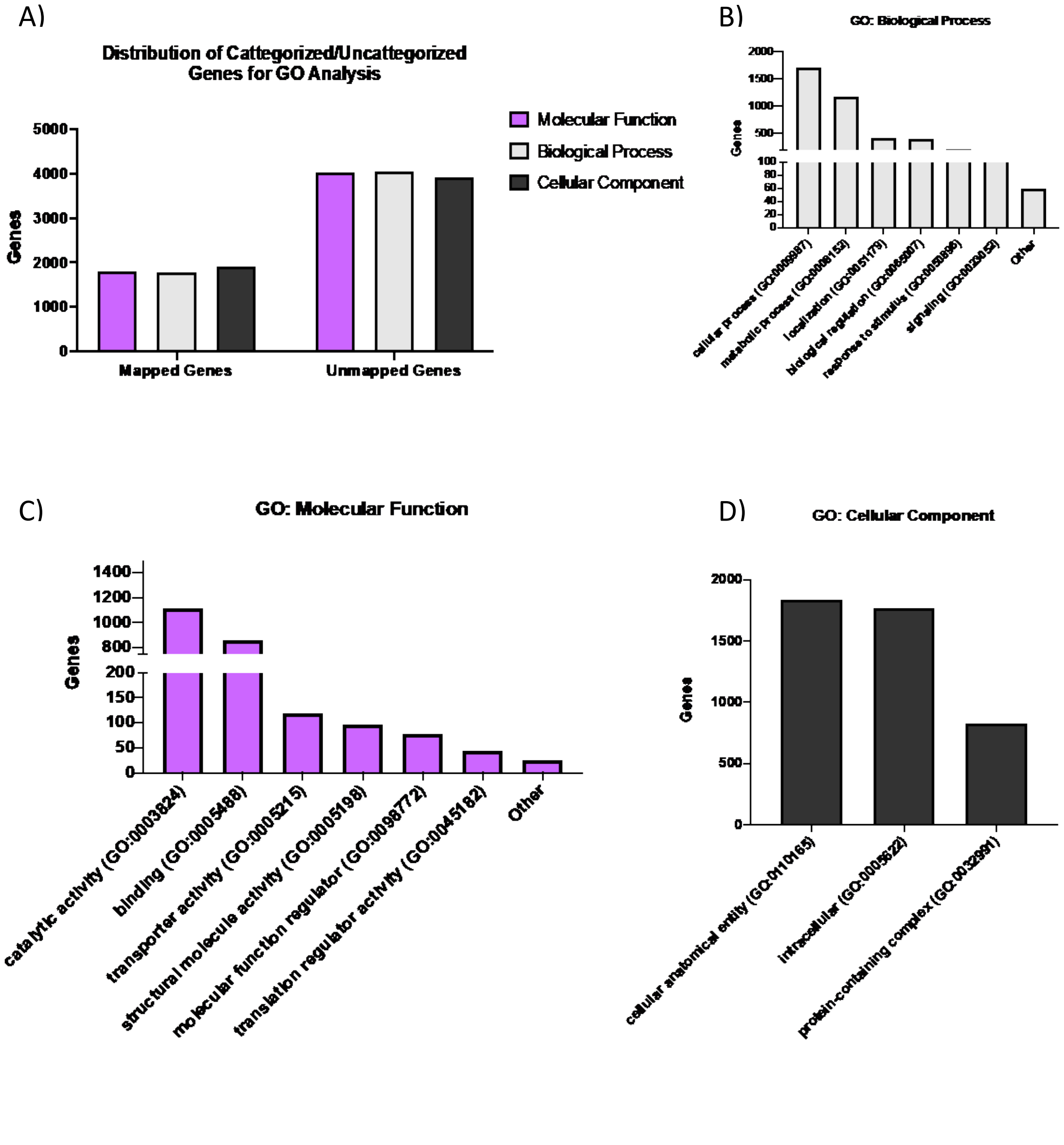

Supplementary Figure 3.

<u>Western blot analysis of SHERP expression in WT and *Δlpg2- L. major*</u>

A) Western blot of 2 and 6 day old culture of WT and Δlpg2- parasites probing for SHERP and H2A loading control. B) Densitometry results from two biological replicates.

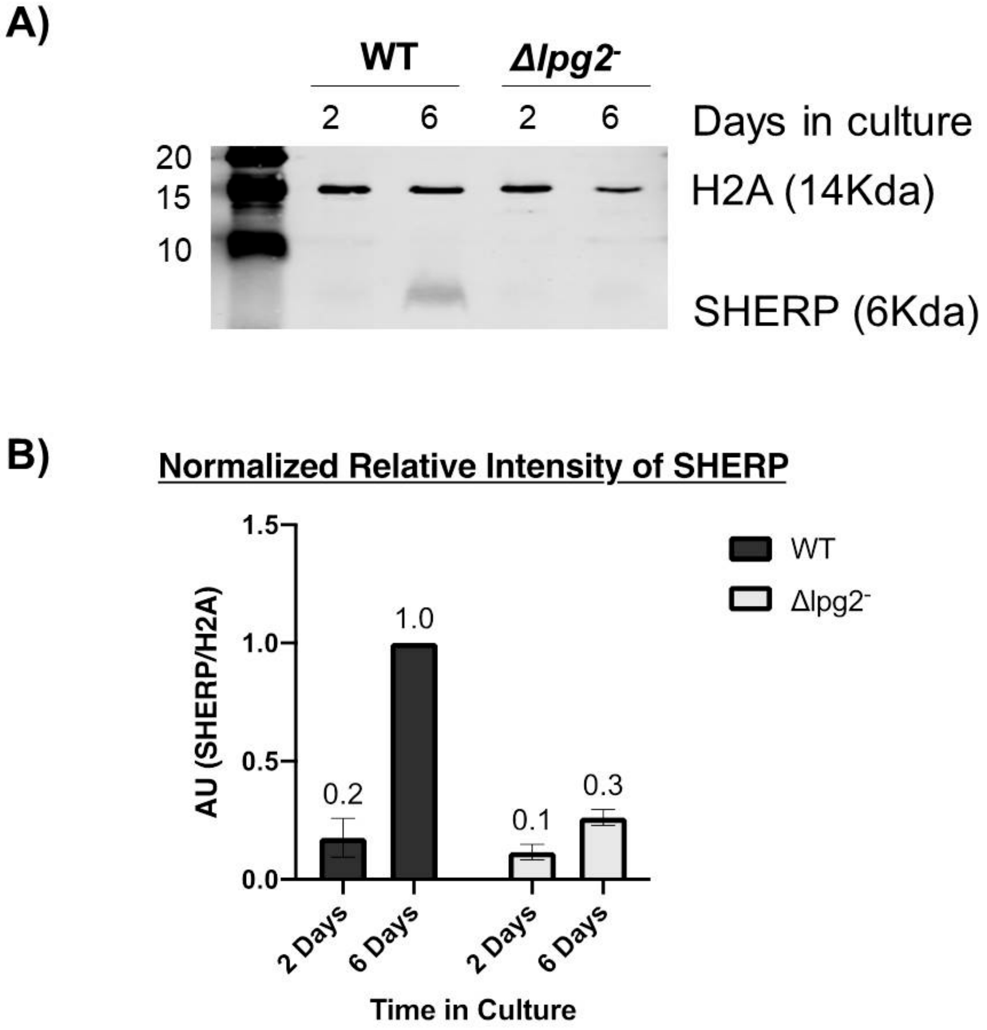

Change Pattern	Protein names	TriTrypDB ID	GO: Cellular Compartment	MiNT DB	log$_2$ Difference of Average LFQ			Corrected ANOVA p-value
					Δlpg2⁻ vs WT	*Δfut1ˢ* vs WT	*Δfut1ˢ* vs *Δlpg2⁻*	
**Down in *Δfut1ˢ***	3-hydroxy-3-methylglutaryl coenzyme A reductase (HMG-CoA reductase) (EC 1.1.1.34)	LmjF.30.3190	Endoplasmic reticulum membrane; Peroxisomal membrane	X	0.87	-1.38	-2.26	0.01
	60S ribosomal export protein NMD3	LmjF.26.0970	Nucleus; cytoplasm		0.37	-1.26	-1.63	0.03
	60S ribosomal protein L11	LmjF.22.0030	Cytosolic large ribosomal subunit		0.83	-1.42	-2.25	0.02
	60S ribosomal protein L18a	LmjF.35.0600	Cytosolic large ribosomal subunit		0.12	-1.28	-1.39	0.05
	Aldehyde dehydrogenase, mitochondrial (EC 1.2.1.3)	LmjF.25.1120	-	X	0.12	-2	-2.13	0.01
	ATP-dependent RNA helicase-like protein	LmjF.36.4400	Nucleolus		0.69	-1.17	-1.87	0.02
	Biopterin transporter BT1 (Biopterin transporter,putativewith=GeneDB:LinJ35_V3.5120)	LmjF.35.5150	-		1.12	-2.03	-3.15	0.01
	Chaperonin HSP60, mitochondrial	LmjF.36.2030	-	X	0.8	-0.81	-1.61	0.04
	Conserved CBS domain protein	LmjF.35.0760	Nucleotide-activated protein kinase complex; Nucleus; Cytoplasm		0.68	-0.81	-1.5	0.05
	Cysteine desulfhydrase (EC 4.4.1.1)	LmjF.32.2640	-		1.03	-2.43	-3.46	0

D-isomer specific 2-hydroxyacid dehydrogenase-like protein;D-isomer specific 2-hydroxyacid dehydrogenase-protein	LmjF.34.1410	-		-0.02	-2.29	-2.27	0.01
Delta-12 fatty acid desaturase	LmjF.33.3270	-		0.44	-2.08	-2.52	0.04
Delta-4 fatty acid desaturase	LmjF.14.1340	-		1.14	-0.53	-1.67	0.05
DNA-directed RNA polymerase subunit beta (EC 2.7.7.6)	LmjF.25.0620	RNA polymerase I complex		0.46	-1.07	-1.53	0.03
Elongation of fatty acids protein (EC 2.3.1.199) (Very-long-chain 3-oxoacyl-CoA synthase)	LmjF.14.0740	Integral component of endoplasmic reticulum membrane		1.66	-0.63	-2.28	0.02
eukaryotic translation release factor, putative	LmjF.27.1710	-		0.19	-1.71	-1.9	0.05
Fibrillarin	LmjF.36.3070	Small-subunit processome; Box C/D snoRNP complex; Cajal body		0.69	-1.98	-2.68	0.01
Glucose transporter, lmgt2	LmjF.36.6290	-		0.09	-1.7	-1.79	0.03
Kinetoplast-associated protein-like protein	LmjF.27.0240	-	X	1.6	-3.8	-5.4	0
Membrane-bound acid phosphatase 2	LmjF.23.1170	-		0.32	-1.66	-1.98	0.05
Metallo-peptidase, Clan ME, Family M16 (EC 1.10.2.2)	LmjF.35.1380	Mitochondrion	X	0.26	-1.14	-1.4	0.04
Methionine synthase (EC 2.1.1.13) (5-methyltetrahydrofolate--homocysteine methyltransferase)	LmjF.07.0090	Cytosol		0.31	-1.89	-2.2	0.01
Methyltransferase-like protein (EC 2.1.1.-)	LmjF.34.2750	Nucleolus		1.18	-1.25	-2.43	0.01

Nucleobase transporter	LmjF.11.0550	Integral component of membrane; Plasma membrane		2.16	-2.1	-4.26	0
Nucleolar GTP-binding protein 1	LmjF.33.1870	-		0.71	-0.75	-1.46	0.04
Periodic tryptophan protein 2-like protein	LmjF.18.0830	Pwp2p-containing subcomplex of 90S preribosome; Small-subunit processome		1.23	-0.55	-1.78	0.03
Phosphotransferase (EC 2.7.1.-)	LmjF.21.0240	Cytosol; Mitochondrion	X	0.24	-1.5	-1.74	0.04
Proline dehydrogenase (EC 1.5.5.2)	LmjF.26.1610	Mitochondrion	X	1.34	-0.07	-1.4	0.02
Proteasome subunit alpha type (EC 3.4.25.1)	LmjF.14.0310	Proteasome core complex, alpha-subunit complex; Nucleus; Cytoplasm; Proteasome core complex		-0.23	-1.85	-1.62	0.04
Putative 3'-nucleotidase/nuclease	LmjF.12.0400	-		1.03	-1.21	-2.24	0.01
Putative 3'-nucleotidase/nuclease (EC 3.1.30.1)	LmjF.31.2310	-		0.71	-2.72	-3.43	0.01
Putative 60S ribosomal protein L21	LmjF.16.0460	Cytosolic large ribosomal subunit		-0.88	-3.09	-2.21	0.04
Putative asparaginyl-tRNA synthetase (EC 6.1.1.22)	LmjF.34.2340	-	X	0.42	-1.06	-1.48	0.03
Putative ATP-binding cassette protein subfamily F,member 2	LmjF.19.0800	-		0.22	-1.33	-1.55	0.04
Putative ATP-dependent RNA	LmjF.30.3250	Nucleolus;		0.92	-0.66	-1.57	0.05

helicase		Intracellular anatomical structure					
Putative ATP-dependent RNA helicase	LmjF.34.2050	Nucleolus;		0.81	-1	-1.81	0.03
Putative beta-fructofuranosidase (EC 3.2.1.26)	LmjF.04.0310	-		4.03	-1.91	-5.94	0
Putative carbamoyl-phosphate synthase (EC 6.3.4.16)	LmjF.16.0590	Cytoplasm	X	0.56	-0.69	-1.25	0.05
Putative chaperone protein DNAj	LmjF.18.1490	Nucleus; cytosol		-0.02	-2.1	-2.08	0.05
Putative cytochrome c oxidase VIII (COX VIII) (EC 1.9.3.1)	LmjF.31.1570	-	X	0.63	-2.16	-2.79	0.02
Putative DEAD box RNA helicase	LmjF.36.1850	Nucleus		0.13	-1.54	-1.67	0.03
Putative DEAD box RNA helicase	LmjF.36.1840	Nucleolus		0.97	-0.65	-1.62	0.04
Putative DNA polymerase epsilon catalytic subunit (EC 2.7.7.7)	LmjF.35.4360	Epsilon DNA polymerase complex		0.42	-1.48	-1.91	0.03
Putative DNA-directed rna polymerase I largest subunit (EC 2.7.7.6)	LmjF.16.1350	RNA polymerase I complex;	X	0.72	-0.69	-1.42	0.04
Putative eukaryotic initiation factor 4a	LmjF.36.6060	eukaryotic translation initiation factor 4F complex		0.56	-0.75	-1.31	0.04
Putative heat shock protein	LmjF.18.1370	Nucleus; Cytosol	X	0.56	-0.83	-1.38	0.04
Putative membrane-bound acid phosphatase 2	LmjF.36.2590	-		0.34	-2.84	-3.18	0
Putative NADH dehydrogenase (EC 1.6.99.3)	LmjF.36.5380	-	X	0.47	-1.2	-1.67	0.04
Putative nucleoside transporter 1	LmjF.15.1240	Integral component of membrane; Plasma membrane		0.35	-2.16	-2.51	0.02

Putative ornithine decarboxylase (EC 4.1.1.17)	LmjF.12.0280	Cytoplasm	0.71	-1.07	-1.78	0.01
Putative oxidoreductase (EC 1.6.5.5)	LmjF.36.4170	-	0.84	-1.35	-2.19	0.02
Putative pantothenate kinase subunit (EC 2.7.1.33)	LmjF.28.0140	Nucleus; Cytosol	1.58	-0.75	-2.33	0.01
Putative pumilio/PUF RNA binding protein 7	LmjF.32.1750	Cytoplasm	0.56	-1.14	-1.7	0.03
Putative pyruvate/indole-pyruvate carboxylase (EC 4.1.1.74)	LmjF.34.3250	Cytosol	0.94	-1.93	-2.87	0.03
Putative RNA 3'-terminal phosphate cyclase (EC 6.5.1.4)	LmjF.35.1700	Nucleolus	1.42	-1.16	-2.57	0.04
Putative RNA binding protein	LmjF.35.2550	-	0.78	-1.16	-1.94	0.02
Putative RNA-binding protein	LmjF.17.0550	-	0.4	-2.09	-2.5	0.05
Putative RNA-binding protein	LmjF.30.2610	-	0.4	-1.08	-1.48	0.04
Putative rRNA methyltransferase (EC 2.1.1.-) (2'-O-ribose RNA methyltransferase SPB1 homolog)	LmjF.27.1980	Nucleolus; Preribosome, large subunit precursor	1.17	-0.78	-1.95	0.03
Putative serine palmitoyltransferase (EC 2.3.1.50)	LmjF.35.0320	Endoplasmic reticulum membrane; Membrane protein complex; Transferase complex; Vacuole; Plasma membrane; Serine C-palmitoyltransferase complex; Cytoplasm	0.23	-2.2	-2.43	0
Putative small nuclear ribonucleoprotein	LmjF.34.3860	Spliceosomal tri-snRNP complex; U1 snRNP; SMN-	-0.31	-1.85	-1.55	0.04

		Sm protein complex; U2 snRNP; U4 snRNP; pICln-Sm protein complex; catalytic step 2 spliceosome; U5 snRNP; U12-type spliceosomal complex; precatalytic spliceosome; commitment complex					
Putative translation initiation factor	LmjF.17.1290	Eukaryotic translation initiation factor 3 complex	X	0.78	-0.59	-1.36	0.05
Putative U3 small nucleolar ribonucleoprotein protein MPP10	LmjF.29.0750	Mpp10 complex; Small-subunit processome		0.7	-1	-1.69	0.05
Putative Unc104-like kinesin	LmjF.34.4260	Microtubule; Kinesin complex		2.07	-0.4	-2.48	0.01
Quinonoid dihydropteridine reductase (EC 1.5.1.34);Quinonoid dihydropteridine reductase (EC 1.5.1.34) (EC 1.6.99.7)	LmjF.34.4510	Cytoplasm		0.42	-3.1	-3.52	0.04
RNA cytidine acetyltransferase (EC 2.3.1.-) (18S rRNA cytosine acetyltransferase)	LmjF.17.1250	Nucleolus		0.74	-0.68	-1.43	0.04
RNA helicase (EC 3.6.4.13)	LmjF.27.0050	Nucleus		0.74	-0.95	-1.69	0.04
RNA helicase (EC 3.6.4.13)	LmjF.32.2230	Nucleolus		0.69	-0.95	-1.65	0.04
Serine/threonine protein phosphatase-like protein	LmjF.26.2100	-		0.88	-0.97	-1.85	0.05
Thiamine biosynthesis-like	LmjF.33.1680	-		0.5	-1.24	-1.75	0.01

	protein					
	tRNA (adenine(58)-N(1))-methyltransferase non-catalytic subunit TRM6	LmjF.36.5780	Nucleus; tRNA (m1A) methyltransferase complex	0.73	-0.82	-1.54	0.04
	Tyrosine aminotransferase (EC 2.6.1.5)	LmjF.36.2360	-	0.08	-1.25	-1.33	0.04
**Down in *Δfut1*^{S}, variably down in *Δlpg2*$^{-}$**	Adenylate cyclase-like protein (EC 4.6.1.1)	LmjF.28.0090	-	-1.48	-2.88	-1.39	0.02
	As/Sb Reductase (EC 1.20.4.1)	LmjF.32.2740	Nucleus; Cytoplasm	-1.58	-3.21	-1.63	0.04
	Autophagy-related protein	LmjF.19.1630	-	-1.21	-2.92	-1.71	0.04
	Cyclin-dependent kinases regulatory subunit	LmjF.32.3790	Cyclin-dependent protein kinase holoenzyme complex; SCF ubiquitin ligase complex	-0.88	-2.94	-2.07	0.05
	DNA-directed RNA polymerase ii	LmjF.25.1315	RNA polymerase II, Core complex; RNA polymerase I complex; RNA polymerase III complex	-2.57	-3	-0.42	0.03
	DNA-directed RNA polymerase-like protein	LmjF.28.2060	RNA polymerase I complex; ;RNA polymerase III complex	-0.75	-2.85	-2.09	0.04
	Putative 60S ribosomal protein L39	LmjF.29.2370	Cytosolic large ribosomal subunit	-1.46	-4.71	-3.25	0.02

	Putative carboxypeptidase;Putative carboxypeptidase (EC 3.4.17.19)	LmjF.14.0180	-		-2.26	-2.49	-0.24	0.02
	Putative cytochrome c oxidase subunit 10 (EC 1.9.3.1)	LmjF.23.0370	-	X	-1.34	-2.49	-1.15	0.04
	Putative intraflagellar transport (IFT) protein	LmjF.30.2000	-		-1.81	-2.69	-0.88	0.04
	Putative ubiquitin/ribosomal protein S27a	LmjF.36.0600	Nucleus; Cytoplasm		0.23	-1.86	-2.09	0.04
**Up in *Δfut1*^{S}**	ATP-NAD kinase-like protein (EC 2.7.1.23)	LmjF.06.0460	-		0.56	1.61	1.06	0.05
	CDP-diacylglycerol--inositol 3-phosphatidyltransferase (EC 2.7.8.11)	LmjF.26.2480	Golgi apparatus		1.82	3.02	1.2	0.03
	Conserved TLD domain protein	LmjF.03.0450	-		0.53	1.95	1.41	0.02
	Cysteine peptidase A (CPA)	LmjF.19.1420	Extracellular space; lysosome		0.43	1.93	1.5	0.03
	Glycerol-3-phosphate acyl transferase (EC 2.3.1.15)	LmjF.03.0080	Integral component of endoplasmic reticulum membrane		1.04	2.46	1.43	0.01
	GP63, leishmanolysin (EC 3.4.24.3) (EC 3.4.24.36)	LmjF.10.0470	Cytoplasm		0.13	1.67	1.54	0.02
	Histone H2B (Histone H2B variant 2)	LmjF.17.1220	-		-1.14	2.55	3.69	0.01
	Hydrophilic acylated surface protein b	LmjF.23.1070	-		-0.05	4.79	4.84	0
	Inositol phosphosphingolipid phospholipase C-Like	LmjF.08.0200	Membrane; Endoplasmic reticulum; Cell periphery	X	0.09	2	1.91	0.01
	Kinase-like protein	LmjF.03.0610	-		-0.54	1.39	1.93	0.03
	Mannosyltransferase (EC 2.4.1.-)	LmjF.36.1200	Endoplasmic reticulum;		1.88	2.38	0.5	0.02

		Endoplasmic reticulum membrane					
NAD(P)-dependent steroid dehydrogenase-like protein	LmjF.06.0350	-		0	1.37	1.37	0.04
Palmitoyltransferase (EC 2.3.1.225)	LmjF.23.1430	Endoplasmic reticulum; Golgi apparatus		0.74	1.71	0.96	0.04
Phosphoglycan beta 1,2 arabinosyltransferase	LmjF.02.0220	-		-0.5	1.12	1.62	0.04
Phosphoglycan beta 1,3 galactosyltransferase 3	LmjF.02.0010	-		-0.57	1.23	1.79	0.04
Probable methyltransferase BMT2 homolog (EC 2.1.1.-)	LmjF.23.1090	Nucleolus		1.45	2.45	1	0.02
Putative 3,2-trans-enoyl-CoA isomerase mitochondrial (EC 1.1.1.35) (EC 5.3.3.8)	LmjF.31.2330	Mitochondrion	X	-0.79	2.14	2.93	0.04
Putative 5-oxoprolinase (EC 3.5.2.9)	LmjF.18.1040	Cytosol		0.11	2.18	2.07	0.03
Putative 6-phosphofructo-2-kinase/fructose-2,6-bipho sphatase (EC 3.1.3.-)	LmjF.03.0800	Cytosol		0.06	2.31	2.24	0.02
Putative alpha-1,2-mannosyltransferase (EC 2.4.1.131)	LmjF.35.5250	Membrane; Endoplasmic reticulum membrane		1.01	2.26	1.25	0.05
Putative argininosuccinate synthase (EC 6.3.4.5)	LmjF.23.0260	Cytoplasm		-0.21	1.84	2.05	0.02
Putative ATP-binding cassette protein subfamily	LmjF.23.0380	Cytoplasm		-0.11	1.74	1.85	0.02
Putative calcium/potassium channel (CAKC)	LmjF.01.0810	Integral component of membrane; Plasma membrane		1.72	1.54	-0.18	0.03
Putative calpain-like cysteine peptidase	LmjF.27.0500	Cytoplasm		-0.83	1.29	2.12	0.01
Putative lanosterol synthase (EC 5.4.99.8)	LmjF.06.0650	-		0.89	1.57	0.67	0.04

Putative major surface protease gp63 (EC 3.4.24.36)	LmjF.28.0570	Cytoplasm	-0.67	2.68	3.35	0.05
Putative phosphatidylserine synthase (EC 2.7.8.8)	LmjF.14.1200	-	2	2.15	0.15	0.04
Putative pteridine transporter	LmjF.06.1260	-	0.42	3.77	3.35	0
Putative pumillio protein 4	LmjF.12.0380	Cytoplasm	0.47	2.37	1.9	0.01
Putative Qb-SNARE protein	LmjF.21.0050	Endomembrane system; Integral component of membrane; SNARE complex; Endomembrane system; Vacuole; Plasma membrane	-0.25	2.25	2.5	0.01
Putative serine/threonine-protein kinase Nek1 (EC 2.7.11.1)	LmjF.32.0260	-	-0.86	1.42	2.28	0.03
Putative surface antigen protein	LmjF.12.1090	-	-0.12	2.11	2.23	0.02
Putative surface antigen protein 2	LmjF.12.0870	-	-0.61	3.06	3.66	0.01
Putative Unc104-like kinesin	LmjF.33.2560	Microtubule; Kinesin complex	0.64	2.01	1.36	0.04
Surface antigen-like protein	LmjF.04.0190	-	1.02	2.46	1.45	0.05
Surface antigen-like protein	LmjF.05.0900	-	-0.28	3.19	3.47	0.01
Surface antigen-like protein	LmjF.05.1215	-	-0.23	5.57	5.8	0

Table S5: Annotated **Proteins Significantly Affected in *Δfut1*^{s}**. Proteins determined to differ significantly in abundance between the *Δfut1*^{s}*, Δlpg2*$^{-}$*,* and WT parasite lines were evaluated by ANOVA; those proteins within clusters changing significantly in *Δfut1*^{s} are shown here. Overall, there were 279 proteins in clusters where proteins decreased in *Δfut1*^{s} (142 total of which 70 are of unknown function), increased in *Δfut1*^{s} (107 total of which 70 are of unknown function), or decreased in *Δfut1*^{s} and varieable impacted in *Δlpg2-* (30 total of which 19 are of unknown function). Proteins present in the MiNT database are indicated, as are the correspoding cellular component GO terms when available.

Cluster Pattern	Protein names	TriTrypID	GO Terms: Cellular Compartment	Difference log_2(LFQ intensity)			Corrected ANOVA p-value
				Δlpg2⁻ vs WT	*Δfut1ˢ* vs WT	*Δfut1ˢ* vs *Δlpg2⁻*	
**Down in *Δlpg2⁻***	2-aminoethylphosphonate: pyruvateaminotransferase-like protein	LmjF.03.0040	-	-1.48	1.60	3.08	0.00
	2,4-dienoyl-coa reductase-like protein	LmjF.06.0930	-	-0.69	1.15	1.84	0.04
	4-coumarate:coa ligase-like protein (EC 6.2.1.12)	LmjF.19.0985	-	-1.03	0.25	1.28	0.05
	4-coumarate:coa ligase-like protein (EC 6.2.1.12)	LmjF.19.1005	-	-1.37	0.42	1.79	0.03
	5-methyltetrahydropteroyltriglutamate-homocystein e S-methyltransferase (EC 2.1.1.14)	LmjF.31.0010	-	-1.57	0.72	2.29	0.00
	6-phosphofructo-2-kinase/fructose-2,6-biphosphata se-1-like protein (EC 2.7.1.105) (EC 3.1.3.46)	LmjF.26.0310	Cytosol	-1.01	1.73	2.74	0.01
	Acyl carrier protein	LmjF.27.0290	Mitochondrion	-3.25	-1.05	2.20	0.03
	Aminomethyltransferase (EC 2.1.2.10) (Glycine cleavage system T protein)	LmjF.36.3810	Mitochondrion	-2.01	0.04	2.05	0.01
	Ascorbate peroxidase (EC 1.11.1.11)	LmjF.34.0070	-	-0.78	2.54	3.32	0.00

Calpain-like cysteine peptidase, Clan CA, family C2	LmjF.20.1220	-	-1.69	1.96	3.64	0.00
Casein kinase I-like protein	LmjF.27.1780	Nucleus; Cytoplasm	-1.88	1.84	3.73	0.00
Cysteine peptidase B (CPB) (EC 3.4.22.-)	LmjF.07.0550	-	-1.14	0.22	1.36	0.04
D-3-phosphoglycerate dehydrogenase-like protein (EC 1.1.1.95)	LmjF.03.0030	Cytosol	-2.06	0.81	2.87	0.00
D-lactate dehydrogenase-like protein	LmjF.29.0280	Mitochondrion	-1.92	0.05	1.98	0.01
Developmentally regulated phosphoprotein-like protein	LmjF.20.0280	Mitochondrion	-0.92	1.08	2.01	0.03
Flavoprotein subunit-like protein	LmjF.07.0800	-	-2.24	-1.15	1.10	0.00
Glutamate dehydrogenase	LmjF.28.2910	Cytosol	-1.82	1.07	2.89	0.00
Glycine cleavage system H protein	LmjF.35.4720	Mitochondrion; Cytoplasm	-2.17	-0.45	1.72	0.02
Histone H4	LmjF.06.0010	-	-1.43	1.18	2.61	0.02
Histone H4	LmjF.15.0010	-	-2.22	-1.16	1.06	0.04
hypothetical protein, conserved	LmjF.26.0730	-	-0.50	1.42	1.92	0.02
Infective insect stage-specific protein	LmjF.17.0890	-	-3.59	2.29	5.88	0.00
Kinesin-like protein	LmjF.17.0800	Microtubule; Kinesin complex	-3.03	0.21	3.24	0.00

Lipophosphoglycan biosynthetic protein (Lipophosphoglycan biosynthetic protein (Lpg2))	LmjF.34.3120	Golgi apparatus	-2.86	0.41	3.27	0.01
Metallo-peptidase, Clan MA(E), family 32	LmjF.13.0090	-	-2.08	1.19	3.27	0.00
Metallo-peptidase, Clan MA(E), Family M3 (EC 3.4.15.-);Putative dipeptylcarboxypeptidase (Putative peptidyl dipeptidase)	LmjF.27.2660	-	-1.44	-0.09	1.35	0.02
Methylmalonyl-coa epimerase-like protein (EC 5.1.99.1)	LmjF.26.0020	-	-1.99	-0.07	1.91	0.04
Myosin XXI	LmjF.32.3870	Actin cytoskeleton; Vesicle; Cytoplasm	-0.65	1.58	2.23	0.00
N-ethylmaleimide reductase-like protein	LmjF.12.1140	-	-5.27	1.50	6.77	0.00
NADH-dependent fumarate reductase-like protein (EC 1.3.1.6)	LmjF.35.0830	-	-1.08	0.55	1.63	0.03
Oxygen-dependent coproporphyrinogen-III oxidase (Coprogen oxidase) (Coproporphyrinogenase) (EC 1.3.3.3)	LmjF.06.1270	Cytoplasm	-0.73	1.14	1.87	0.02

Phosphoacetylglucosamine mutase (PAGM) (EC 5.4.2.3) (Acetylglucosamine phosphomutase) (N-acetylglucosamine-phosphate mutase)	LmjF.07.0805	-	-2.02	0.54	2.56	0.00
Phosphodiesterase (EC 3.1.4.-)	LmjF.15.1480	-	-1.34	0.10	1.45	0.02
Phosphomannomutase (EC 5.4.2.8)	LmjF.36.1960	Cytosol	-1.76	0.30	2.06	0.02
Promastigote surface antigen protein PSA;Putative surface antigen protein 2	LmjF.12.0765	-	-2.92	1.46	4.37	0.00
Protein disulfide isomerase (EC 5.3.4.1)	LmjF.06.1050	-	-1.27	1.17	2.45	0.05
Protein kinase A catalytic subunit isoform 1;Protein kinase A catalytic subunit isoform 2	LmjF.35.4010	Protein-containing complex; Intracellular	-2.08	0.05	2.13	0.02
Putative acyl-CoA dehydrogenase (EC 1.3.99.3)	LmjF.28.2510	-	-0.62	0.80	1.42	0.05
Putative acyl-coenzyme a dehydrogenase (EC 1.3.99.3)	LmjF.06.0880	Mitochondrion	-0.46	1.27	1.72	0.04
Putative aldose 1-epimerase (EC 5.1.3.3)	LmjF.35.0970	-	-2.03	1.43	3.46	0.00
Putative amino acid transporter aATP11	LmjF.31.0580	-	-1.49	1.07	2.56	0.01

Putative AMP deaminase (EC 3.5.4.6)	LmjF.35.4800	Cytosol	-1.78	-0.55	1.23	0.03
Putative calmodulin-related protein	LmjF.30.3360	-	-1.76	0.31	2.07	0.03
Putative calpain-like cysteine peptidase	LmjF.27.0510	Cytoplasm;	-2.10	-0.11	1.99	0.01
Putative calpain-like cysteine peptidase	LmjF.31.0390	-	-1.32	0.88	2.20	0.02
Putative calpain-like cysteine peptidase	LmjF.34.0280	-	-0.66	1.57	2.23	0.02
Putative calpain-like cysteine peptidase (EC 3.4.22.-)	LmjF.04.0450	-	-1.06	1.06	2.12	0.02
Putative calpain-like cysteine peptidase (EC 3.4.22.-) (EC 3.4.22.33)	LmjF.20.1190	-	-1.22	1.36	2.59	0.00
Putative calpain-like cysteine peptidase (Small myristoylated protein 1)	LmjF.20.1310	-	-0.72	0.87	1.59	0.03
Putative carboxylase (EC 6.4.-.-)	LmjF.01.0050	Mitochondrion	-0.24	1.74	1.98	0.02
Putative centrin	LmjF.32.0660	Centriole; Centrosome	-2.86	-1.94	0.91	0.03
Putative deoxyuridine triphosphatase (dUTPase) (EC 3.6.1.23)	LmjF.06.0560	-	-1.73	0.37	2.10	0.04
Putative dual specificity protein phosphatase (EC 3.1.3.48)	LmjF.34.2190	Cytoplasm	-2.43	-0.25	2.18	0.02

Putative folate/biopterin transporter	LmjF.35.5190	-	-1.64	0.32	1.97	0.01
Putative folate/biopterin transporter	LmjF.10.0380	-	-6.38	0.30	6.68	0.00
Putative folate/biopterin transporter	LmjF.10.0385	-	-1.79	-0.71	1.08	0.03
Putative glutamamyl carboxypeptidase (EC 3.4.-.-)	LmjF.29.1570	-	-1.94	-1.03	0.91	0.03
Putative histone H3 variant	LmjF.19.0630	-	-1.99	2.25	4.24	0.02
Putative lipase (EC 3.1.1.3)	LmjF.31.2460	-	-1.37	0.97	2.34	0.04
Putative methylenetetrahydrofolate reductase (EC 1.5.1.20)	LmjF.36.6390	Cytosol	-1.84	1.91	3.75	0.01
Putative mitotubule-associated protein Gb4	LmjF.26.1950	Dynein complex; Axoneme	-0.39	1.02	1.41	0.03
Putative N-acyl-L-amino acid amidohydrolase (EC 3.5.1.14)	LmjF.31.1130	-	-1.55	-0.50	1.05	0.04
Putative paraflagellar rod component par4	LmjF.05.0040	-	-2.07	-0.43	1.65	0.04
Putative propionyl-coa carboxylase beta chain (EC 6.4.1.3)	LmjF.28.0490	-	-0.73	1.44	2.17	0.01
Putative ribulose-phosphate 3-epimerase (EC 5.1.3.1)	LmjF.35.3680	Cytosol	-2.67	-1.16	1.51	0.04
Putative serine/threonine protein kinase	LmjF.32.0810	-	-0.72	1.90	2.63	0.01

Putative serine/threonine-protein kinase (EC 2.7.11.1)	LmjF.31.2960	-	-1.41	1.03	2.43	0.01
Putative short chain 3-hydroxyacyl-CoA dehydrogenase (EC 1.1.1.35)	LmjF.36.1140	-	-1.01	0.60	1.62	0.03
Putative small myristoylated protein 4 (Small myristoylated protein 3)	LmjF.20.1280	-	-1.86	-0.21	1.65	0.03
Putative surface antigen protein	LmjF.12.0740	-	-2.22	2.71	4.93	0.00
Putative surface antigen protein 2	LmjF.12.0755	-	-0.96	1.80	2.77	0.01
Putative surface antigen protein 2	LmjF.12.0760	Membrane	-1.30	1.87	3.16	0.00
Putative surface antigen protein 2	LmjF.12.0850	-	-0.95	1.65	2.60	0.01
Putative thymine-7-hydroxylase (EC 1.14.11.6)	LmjF.29.0250	-	-3.00	-1.31	1.69	0.01
Receptor-type adenylate cyclase a (EC 4.6.1.1)	LmjF.17.0200	-	-2.15	-0.90	1.24	0.03
Receptor-type adenylate cyclase a-like protein (EC 4.6.1.1)	LmjF.36.3180	-	-2.51	0.46	2.97	0.00
Ribonuclease mar1	LmjF.12.0060	-	-2.28	0.44	2.72	0.04
Selenoprotein T, putative	LmjF.35.1120	-	-0.50	1.62	2.13	0.03

	Serine/threonine-protein phosphatase (EC 3.1.3.16)	LmjF.26.2530	Protein serine/threonine phosphatase complex; Cytoplasm; Calcineurin complex; Cytoplasm	-1.24	1.00	2.24	0.04
	SHERP (Small hydrophilic endoplasmic reticulum-associated protein (Sherp))	LmjF.23.1086	-	-7.83	0.90	8.73	0.00
	Succinyl-CoA:3-ketoacid-coenzyme A transferase (EC 2.8.3.5)	LmjF.33.2340	-	-1.01	1.02	2.03	0.02
	tb-292 membrane associated protein-like protein (pseudogene)	LmjF.15.0440	-	-1.25	1.98	3.23	0.00
	Triosephosphate isomerase (EC 5.3.1.1)	LmjF.24.0850	Cytosol	-0.76	0.89	1.65	0.04
	Ubiquitin-conjugating enzyme-like protein	LmjF.22.0610	-	-2.64	-0.59	2.05	0.04
**Up in *Δlpg2*⁻**							
	Elongation of fatty acids protein (EC 2.3.1.199) (Very-long-chain 3-oxoacyl-CoA synthase)	LmjF.14.0705	Integral component of endoplasmic reticulum membrane; vacuole; plasma membrane	1.36	0.09	-1.27	0.05
	GP63, leishmanolysin (EC 3.4.24.36)	LmjF.10.0460	Cytoplasm	2.21	0.51	-1.70	0.01
	MGT2 magnesium transporter	LmjF.25.1090	-	1.69	-0.08	-1.77	0.04
	Phosphoglycan beta 1,2 arabinosyltransferase,(SCA like)	LmjF.34.0510	-	3.98	2.27	-1.71	0.00
	Putative 60S ribosomal protein L12	LmjF.35.2190	Large ribosomal subunit; Cytosolic large ribosomal	4.35	-1.91	-6.27	0.03

			subunit				
	Putative amino acid transporter	LmjF.10.0720	Vacuolar membrane	1.83	0.78	-1.05	0.04
	Sucrose-6-phosphate hydrolase (EC 3.2.1.26)	LmjF.23.0880	-	1.92	0.83	-1.09	0.03
**Down in *Δfut1^s^* Variably down in *Δlpg2^-^***	Adenylate cyclase-like protein (EC 4.6.1.1)	LmjF.28.0090	-	-2.13	-2.28	-0.15	0.03
	As/Sb Reductase (EC 1.20.4.1)	LmjF.32.2740	Nucleus; Cytoplasm	-1.58	-3.21	-1.63	0.04
	Autophagy-related protein	LmjF.19.1630	-	-1.48	-2.88	-1.39	0.02
	Cyclin-dependent kinases regulatory subunit	LmjF.32.3790	Cyclin-dependent protein kinase holoenzyme complex; SCF ubiquitin ligase complex	-2.57	-2.85	-0.28	0.01
	DNA-directed RNA polymerase ii	LmjF.25.1315	RNA polymerase II, core complex; RNA polymerase I complex; RNA polymerase III complex	-1.38	-3.13	-1.75	0.03
	DNA-directed RNA polymerase-like protein	LmjF.28.2060	RNA polymerase I complex; RNA polymerase III complex	-1.21	-2.92	-1.71	0.04
	Putative 60S ribosomal protein L39	LmjF.29.2370	Cytosolic large ribosomal subunit	-2.45	-3.75	-1.3	0.02
	Putative carboxypeptidase;Putative carboxypeptidase (EC 3.4.17.19)	LmjF.14.0180	-	-1.89	-2.74	-0.86	0.04
	Putative cytochrome c oxidase subunit 10 (EC 1.9.3.1)	LmjF.23.0370	-	-1.48	-3.45	-1.97	0.02
	Putative intraflagellar transport (IFT) protein	LmjF.30.2000	-	-2.57	-3	-0.42	0.03
	Putative ubiquitin/ribosomal protein S27a	LmjF.36.0600	Nucleus; Cytoplasm	-2.26	-5.68	-3.43	0

Table S6: Proteins Significantly Affected in *Δlpg2⁻*. Proteins differing significantly in abundance between the *Δfut1^s^*, *Δlpg2⁻*, and WT parasite lines were evaluated by ANOVA; those proteins within clusters changing in *Δlpg2⁻* are shown (Fig. 4; Table S3). Overall, there were 174 proteins significantly decreased in *Δlpg2⁻*, of which 89 are of unknown function, while 13 increased in *Δlpg2⁻* of which 6 are of unknown function. A third group of 30 proteins, of which 19 are of unknown function, were significantly decreased in *Δfut1^s^* but varied among the biological replicates in *Δlpg2⁻* (Table S5).
